# Single-cell RNA-seq reveals keratinocyte and fibroblast heterogeneity and their crosstalk via epithelial-mesenchymal transition in psoriasis

**DOI:** 10.1038/s41419-024-06583-z

**Published:** 2024-03-12

**Authors:** Dianhao Guo, Xiaokang Li, Jing Wang, Xin Liu, Yibo Wang, Shuhong Huang, Ningning Dang

**Affiliations:** 1 Department of Dermatology, Shandong Provincial Hospital Affiliated to Shandong First Medical University, Jinan, Shandong China; 2 Department of Biochemistry and Molecular Biology, School of Clinical and Basic Medical Sciences, Shandong First Medical University& Shandong Academy of Medical Sciences, Jinan, Shandong China; 3 Department of Dermatology, Binzhou Medical University Hospital, Binzhou, China; 4 Institute of Basic Medicine, Shandong Provincial Hospital Affiliated to Shandong First Medical University, Jinan, China

**Keywords:** Psoriasis, Psoriasis

## Abstract

The pathogenesis of psoriasis, a chronic inflammatory autoimmune skin disease with a high global prevalence, remains unclear. We performed a high-resolution single-cell RNA sequencing analysis of 94,759 cells from 13 samples, including those from psoriasis model mice and wild-type mice. We presented a single-cell atlas of the skin of imiquimod-induced mice with psoriasis and WT mice, especially the heterogeneity of keratinocytes and fibroblasts. More interestingly, we discovered that special keratinocyte subtypes and fibroblast subtypes could interact with each other through epithelial–mesenchymal transition and validated the results with drug verification. Moreover, we conducted a tentative exploration of the potential pathways involved and revealed that the IL-17 signalling pathway may be the most relevant pathway. Collectively, we revealed the full-cycle landscape of key cells associated with psoriasis and provided a more comprehensive understanding of the pathogenesis of psoriasis.

## Introduction

Psoriasis is a chronic inflammatory autoimmune skin disease with a high global prevalence of 2–3% [1] that is mediated by innate and adaptive immune systems [2]. Various cells, including keratinocytes (KCs), fibroblasts (FBs), plasmacytoid dendritic cells (pDCs), and macrophages, as well as multiple cytokines, such as TNF-α, IL-12 and IL-17A, are involved in the entire immune process [3, 4]. Although the pathogenesis of psoriasis has been investigated extensively, the full-cycle landscape of key cells associated with psoriasis remains unclear.

KCs are key cells in the pathogenesis of psoriasis. They play an important role in resisting environmental damage and maintaining skin homeostasis [5]. Various factors, such as genetic regulation, cytokines, receptors, and transcription factors, regulate keratinocytes in both the initiation and maintenance phases of psoriasis [6]. To date, how various factors work together to regulate the functions of keratinocytes remains unclear.

Recently, increasing attention has been given to the role of FBs in psoriasis. Skin FBs are closely associated with the condition and function of the skin by producing collagen and other intercellular matrix substances [7]. Gęgotek et al. [8] reported that dermal FBs in patients with psoriasis were dysfunctional, possibly due to upregulation of proinflammatory factors, antioxidant proteins and factors related to signal transduction and proteolytic processes. Moreover, the downregulated proteins in psoriatic FBs were mostly associated with transcriptional/translational processes, glycolysis/adenosine triphosphate synthesis and structural molecules. Consequently, epidermal cells such as KCs were induced to hyperproliferation due to FB dysfunction. Obviously, FBs and KCs interact with each other in the occurrence and development of psoriasis. However, the detailed intercellular signalling is unclear.

Epithelial–mesenchymal transition (EMT) is a series of transformation processes in which epithelial cells are transformed from an adhesive cell to a free cell with a mesenchymal phenotype [9]. EMT and the reverse process mesenchymal-epithelial transition (MET) occur during the entire course of embryonic development and finally promote tissue morphogenesis [10]. Following trauma and inflammatory injury, mature epithelial or endothelial cells in the tissue transform into FBs and other related cells, leading to tissue remodelling [11]. In the last few decades, EMT has been identified in diverse cancers, including breast cancer [12], head and neck cancer [13], uveal melanoma [14], and colorectal cancer [15]. However, few studies have investigated the EMT phenomenon in psoriasis.

Single-cell RNA sequencing (scRNA-seq) is a powerful tool to detect cell specificity and intercell differences, explore the cooperative mode of intercell operation from the perspective of cell mapping. As a newly developed technology, scRNA-seq has been widely applied in inflammatory skin diseases such as psoriasis [16]. Chenxin Qie et al. revealed the transcriptional landscape and heterogeneity of skin macrophages in Vsir knockout murine psoriasis with scRNA-seq analysis [17]. Jared Liu et al. performed scRNA-seq of psoriatic and healthy skin and disclosed the different landscape of CD8+ T cells [18]. In 2021, Gary Reynolds et al. used scRNA-seq to uncover the cellular heterogeneity and organization of human first-trimester prenatal skin, adult healthy skin and skin of patients with AD and psoriasis [19]. ScRNA-seq enables precise analysis of single-cell sequencing, greatly improving the resolution of cell heterogeneity studies, as well as signalling pathways.

As psoriasis is a complicated skin disease, choosing the right animal model is particularly important. The psoriasis mouse model induced by imiquimod has become the most commonly used animal model of psoriasis due to its simplicity, convenience and similarities with psoriasis in pathological characteristics [20]. At present, different drugs, such as the biological agents secukinumab (SEC), ustekinumab (UST) and adalimumab (ADA), are used to treat psoriasis. Novel small molecule inhibitors, such as SHP099, are also being explored. As a potent allosteric inhibitor of SHP2, SHP099 ameliorates IMQ-induced psoriatic development in mice through an unclear molecular mechanism. To address this question, researchers used scRNA-seq to explore the role of SHP2 in all cell types in IMQ-induced mouse skin upon SHP099 treatment [21, 22]. Our previous study showed that *Chrna*5 influences psoriasis-related inflammation and terminal skin differentiation. *Chrna5* knockout (KO) inhibited epidermal proliferation and inflammation in mice. Moreover, scRNA-seq analysis revealed that *Chrna5* KO significantly reduced the number of KCs [23]. Therefore, the *Chrna5* KO mouse model provides a good model to study the role of KCs in psoriasis.

In this study, to research the critical role of KCs and FBs as well as their dynamic crosstalk through EMT in psoriasis, we performed scRNA-seq analysis combined with bulk RNA-seq, gene array and experimental verification to obtain a comprehensive understanding of the pathogenesis of psoriasis.

## Results

### Single-cell atlas of the skin of mice with psoriasis and WT mice

**Fig. 1:**
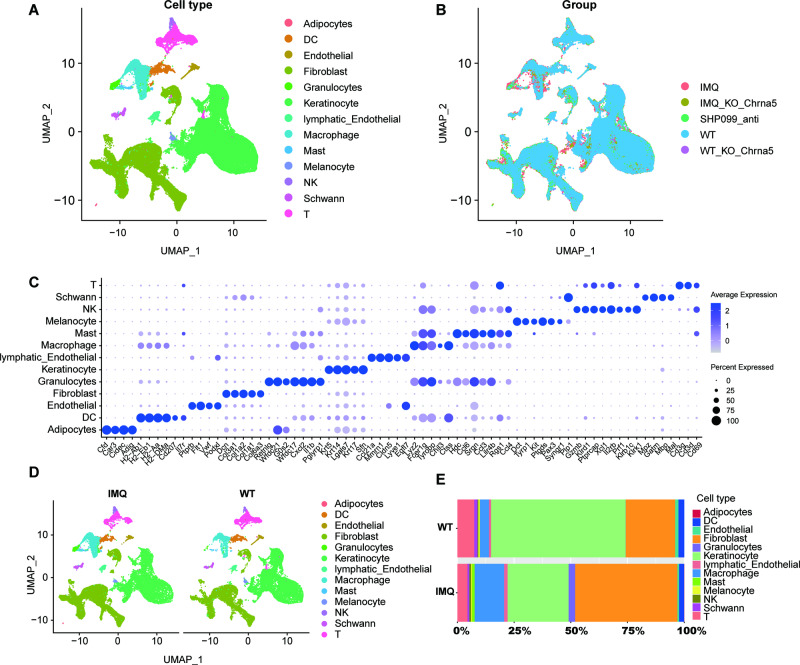
Single-cell atlas of the skin of mice with psoriasis and WT mice. UMAP plot of the 94,759 cells from 13 samples in the skin of mice, coloured by cell Type (**A**), and group (**B**). Each dot denotes a single cell. **C** Dot plot of the expression levels of marker genes for cell types defined in **A**. Dot size corresponds to the percent of expressing cells, and dot colour indicates the average expression levels. **D** UMAP plot of the cells from the skin of the IMQ group and WT group, coloured by cell type. **E** Proportions of cell types in the skin of the IMQ group and WT group.

For identification of the cell composition of psoriatic skin, 13 samples were collected in this study, 4 imiquimod-induced psoriatic mouse samples (IMQ group), 5 wild-type mouse samples (WT group), 2 mouse samples that were induced by imiquimod and treated with SHP099 hydrochloride for an additional 4 days (SHP099 group), 1 Chrna5 knockout mouse sample (KO group), and 1 Chrna5 knockout imiquimod-induced psoriasis sample (KO IMQ group). (For details, please see the Materials and Methods) (Fig. 1A, B; Fig. S1A; Supplementary Table 1). With prior assessment, 94 759 cells were screened, and 13 cell populations were identified: adipocytes (47), DCs (1 810), endothelial cells (911), FBs (33 118), granulocytes (1 067), KCs (42 073), lymphatic endothelial cells (1 156), macrophages (6 656), mast cells (444), melanocytes (532), NK cells (547), Schwann cells (997), and T cells (5 401) (Fig. 1A; Fig. S1B, C). The expression levels of marker genes showed that cell subpopulations were well annotated (Fig. 1C). Then we extracted samples from the total cell population of the IMQ and WT groups and compared the changes in cell proportions between the two groups to find out the key cell subpopulations in the pathogenesis of psoriasis. (Fig. 1D). KCs, FBs, macrophages and T cells were the main populations that differed between the IMQ group and the WT group (Fig. 1E).

### KC heterogeneity in psoriasis

**Fig. 2:**
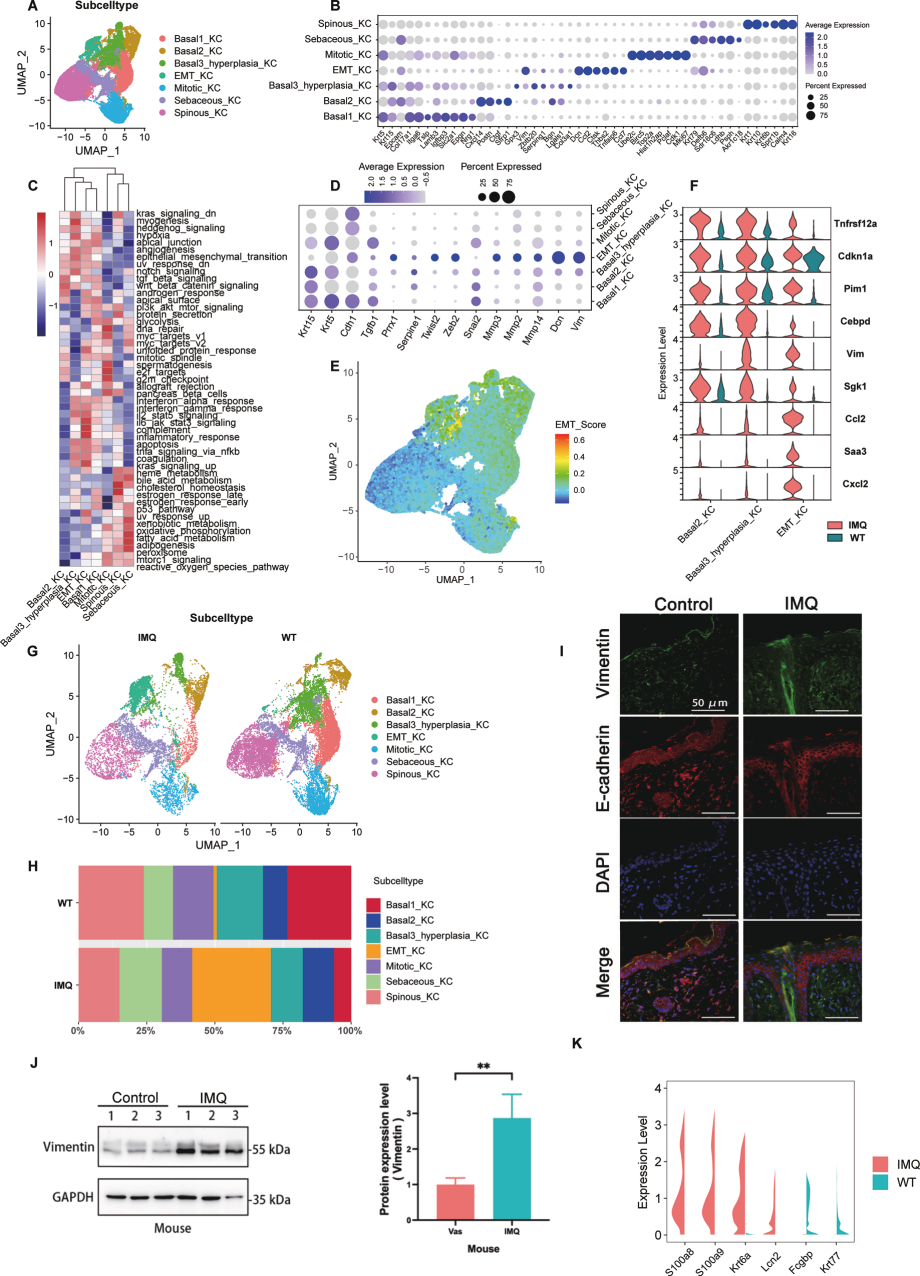
KC heterogeneity in psoriasis. **A** UMAP plot of 42,073 KCs coloured by cell subtype. Each dot denotes a single cell. **B** Dot plot of the expression levels of marker genes for keratinocyte subtypes defined in **A**. Dot size corresponds to the percent of expressing cells, and dot colour indicates the average expression levels. **C** Heatmap showing differences in hallmark pathway activities scored per cell with GSVA among different keratinocyte subtypes. **D** Dot plot of the expression levels of marker genes for EMT in all keratinocyte subtypes defined in **A**. Dot size corresponds to the percent of expressing cells, and dot colour indicates the average expression levels. **E** The distribution of signature scores of 194 EMT genes among all keratinocyte subtypes defined in **A**. **F** Violin plots showing the top differentially expressed genes (DEGs) of the EMT KC subset in the IMQ group compared with the WT group (red: IMQ; green: WT). **G** UMAP plot of all keratinocyte subtypes from the skin of the IMQ group and WT group, coloured by cell type. **H** Proportions of keratinocyte subtypes in the skin of the IMQ group and WT group. **I** Fluorescence immunohistochemistry of EMT associated proteins in mouse skin of the IMQ group and WT group. **J** WB of EMT associated proteins in mouse skin of the IMQ group and WT group. **K** Violin plots illustrating the DEGs of KCs in the IMQ group and WT group.

Further analyses were conducted to better interpret the heterogeneity of KCs. 7 KC subtypes (basal 1 KC, basal 2 KC, basal 3 hyperplasia KC, EMT KC, mitotic KC, sebaceous KC and spinous KC) were identified based on marker gene expression (Fig. 2A, B; Fig. S2A, B). The results of the gene set variation analysis (GSVA) and marker genes suggested that basal 2 KC, basal 3 hyperplasia KC and EMT KC exhibit a high level of EMT (Fig. 2C). Gene expression profiles showed that EMT-related marker genes, such as *Vim*, *Dcn*, *Mmp*, *Zeb2* and *Twist2*, were especially high in EMT KCs (Fig. 2D). The EMT score of the KC subtype demonstrated that basal 2 KC, basal 3 hyperplasia KC and EMT KC had high EMT scores compared with other KC subtypes (Fig. 2E; Fig. S2C). The top differentially expressed genes (DEGs) of the EMT KC subtype in the IMQ group compared with the WT group were then further dissected (Fig. 2F). Most of the DEGs, such as *Vim*, *Saa3* and *Cebpd*, were EMT-associated genes, which further suggested that the EMT phenomenon was more prevalent in the EMT KCs in the IMQ group than those in the WT group.

To further prove this point, we extracted all KCs from the IMQ and WT groups to compare the differences in cell proportions between each KC subpopulation (Fig. 2G, Fig. S2D). The comparison revealed that the proportion of EMT KC subtypes increased in the IMQ group (Fig. 2H). To verify the results, we performed a fluorescence immunohistochemistry experiment with the skin of mice in the IMQ and control groups (treated with Vaseline) (Fig. 2I). The results showed that the expression of *Vimentin* (*Vim*) was stronger, and the expression of E-cadherin was decreased in the IMQ group compared with control group, which indicated a greater level of EMT in the IMQ group than in the control group. To further confirm the result of the fluorescent immunohistochemistry experiment, we performed western blotting (WB) to obtain quantitative results. As shown in Fig. J, the WB results showed an 80% increase in *Vim* expression in the IMQ group compared with the control group.

The top DEGs of the IMQ group compared with the WT group were analysed with bulk RNA-seq and scRNA-seq analysis to explore the potential signalling pathway related to the EMT process. (Fig. 2K; Fig. S2E, F). The expression of *S100a8* and *S100a9* was increased in IMQ-induced psoriasis both in bulk RNA-seq and scRNA-seq. *S100a8* and *S100a9* are genes related to the IL-17 signalling pathway. Here, we speculated that the IL-17 signalling pathway may be the main pathway regulating the EMT process.

### FB heterogeneity in psoriasis

**Fig. 3:**
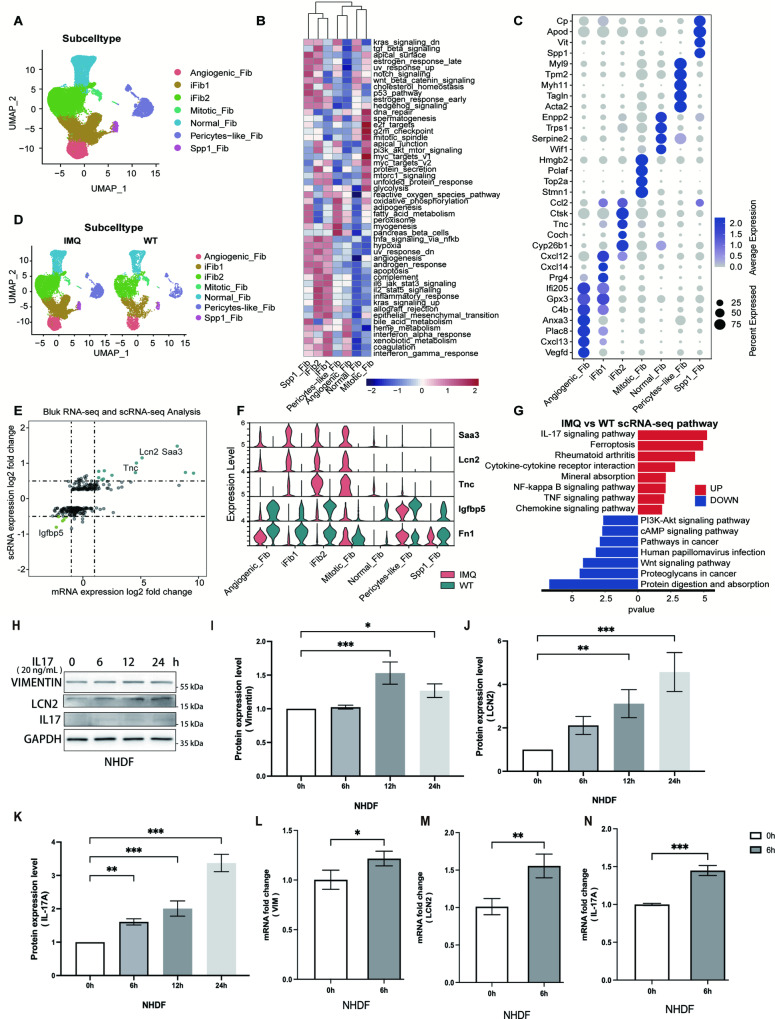
FB heterogeneity in psoriasis. **A** UMAP plot of 33,118 FBs coloured by cell subtype. Each dot denotes a single cell. **B** Heatmap showing differences in hallmark pathway activities scored per cell with GSVA among different fibroblast subtypes. (**C**) Dot plot of the expression levels of marker genes for FB subtypes defined in **A**. Dot size corresponds to the percent of expressing cells, and dot colour indicates the average expression levels. **D** UMAP plot of all fibroblast subtypes from the skin of the IMQ group and WT group, coloured by cell type. **E** Bulk RNA-seq and scRNA-seq analysis of the DEGs in the IMQ group and WT group (bulk RNA-seq data source GSM2299980 (WT), GSM2299981 (WT), GSM229998 (IMQ), GSM2299983 (IMQ)). **F** Violin plots showing the top DEGs between IMQ and WT in all fibroblast subtypes. **G** KEGG pathway map of IMQ vs. WT scRNA-seq in FBs. **H** Western blot image of Vim, Lcn2 and IL-17 expressions in HaCaT cells treated with IL-17 for indicated time. **I**–**K** Histogram of protein expression level in **H**. **L**–**N** Quantification of *Vim, Lcn2 and IL-17* mRNA expressions in NHDF cells stimulated with IL-17 (20 ng/mL) for 6 h.

In this study, 7 FB subtypes were recognized: angiogenic fibroblasts (Angiogeni_Fib), inflammation-associated fibroblasts (iFib1, 2), mitotic fibroblasts (Mitotic_Fib), normal fibroblasts (Normal_Fib), pericyte fibroblasts (Pericytes_Fib) and secreted phosphoprotein 1 fibroblasts (Spp1_Fib) (Fig. 3A–C; Fig. S3A, B). IFib1 and iFib2 were the two largest clusters among the FB subtypes (Fig. 3A). Compared to that of the WT group, the proportion of iFib1 and iFib2 increased obviously in the IMQ group (Fig. 3D; Fig. S3C). This finding indicated that iFibs might play an important role in psoriasis. To confirm this result, we performed combined bulk RNA-seq and scRNA-seq analysis between the IMQ group and the WT group. The inflammation-specific markers *Saa3* and *Lcn2* were the genes that differed the most between the two groups (Fig. 3E, F; Fig. S3D). Studies have shown that *Lcn2* expression is related to EMT [24]. Moreover, the difference was notable in iFib1, iFib2 and Mitotic_Fib, which further demonstrated that iFib was associated with the process of psoriasis.

Various FB subtypes were involved in different signalling pathways. As shown in Fig. 3B, in addition to the inflammatory response, iFib1 and iFib2 also showed high levels of EMT in the GSVA results. The KEGG pathway map of IMQ vs. WT scRNA-seq showed that the IL-17 signalling pathway was the most significantly enriched pathway (Fig. 3G). Combined with the EMT phenomenon in KCs and its possible relationship with the IL-17 signalling pathway, the results indicated that iFib may interact with KCs through EMT and that the IL-17 signalling pathway may be the most relevant pathway. So, we performed in vitro experiments to confirm whether IL-17 signalling could modulate the EMT process in fibroblasts. First, we treated the primary normal human dermal fibroblast (NHDF) with IL-17 (20 ng/ml) and detected the expression of EMT-related proteins, Vim and Lcn2 by western blotting. The results showed that IL-17 upregulated the protein level of Vim and Lcn2 significantly 12 h after treatment (Fig. 3H–J). Moreover, we found that IL-17 can trigger the expression of endogenous IL-17(Fig. 3K). This result suggests the possibility of a potential positive feedback loop of IL17. Further, we confirmed that the upregulation of mRNA occurs within 6 h, indicating that the upregulation of Vim and Lcn2 occurs at the transcriptional level, consistent with single-cell sequencing results (Fig. 3L–N). Therefore, our results suggest that IL-17 is the main inducer of the EMT process in fibroblasts.

### Dynamic evolution process of EMT in psoriasis

**Fig. 4:**
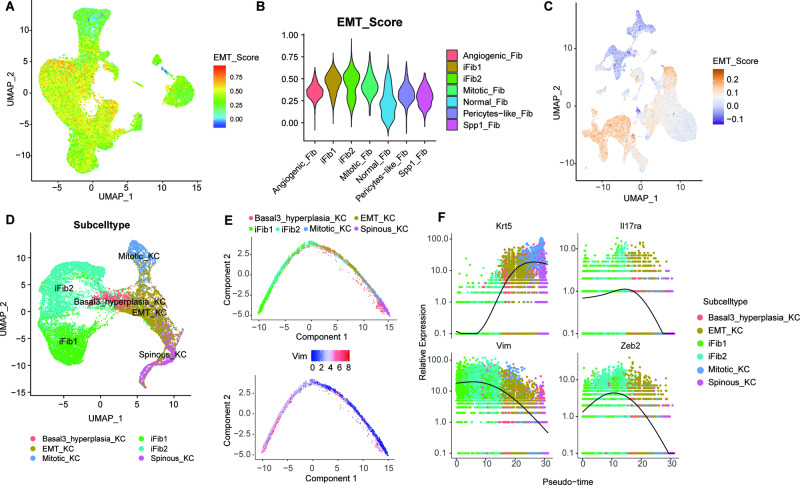
Dynamic evolution process of EMT in psoriasis. **A** UMAP of 33118 FBs showing the EMT score. **(B)** Violin plot showing the EMT score of FBs, coloured by fibroblast subtypes. **C** UMAP plot of the 94,759 cells from 13 samples in the skin of mice, showing the EMT score. **D** Recluster and UMAP visualization of EMT-related subtype, including iFib1, iFib2, basal 3 hyperplasia KCs, EMT KCs, Mitotic_KCs and Spinous_KCs. Each dot denotes a single cell. **E** The EMT progress between fibroblasts and keratinocytes. **F** Temporal variation in an IL-17 signalling pathway marker (*Il17ra*), keratinocyte markers (*Krt5*), and EMT markers (*Vim* and *Zeb2*) in EMT-related subtypes. Each point indicates a single cell, coloured by cell subtype.

The dynamic evolution process of EMT in psoriasis were explored extensively to verify the aforementioned speculation. The EMT subpopulation of FBs was first reconfirmed (Fig. 4A, B; Fig. S4). IFib1 and iFib2 had the highest EMT scores, which is consistent with a previous study. The EMT score of the 94 759 cells from all 13 samples validated that the FB and KC clusters were the main cell types correlated with the EMT ≈ (Fig. 4C). Subsequently, reclustering and UMAP visualization of EMT populations, including iFib1, iFib2, basal 3 hyperplasia KCs, EMT KCs, Mitotic_KCs and Spinous_KCs, were conducted (Fig. 4D). A diffusion map and principal component analysis showed their developmental trajectories, which were sequential processes from Spinous_KC to Mitotic_KC, basal 3 hyperplasia KC, EMT KC, iFib2 and iFib1 (Fig. 4E). Furthermore, as the KC marker *Krt5* increased, the EMT markers *Vim* and *Zeb2*, and the IL-17 signalling pathway marker *Il17ra* clearly decreased, which hinted at the existence of EMT in psoriasis and its intimate connection with the IL-17 signalling pathway (Fig. 4F).

### *Chrna5* KO reduces EMT KCs in psoriasis

**Fig. 5:**
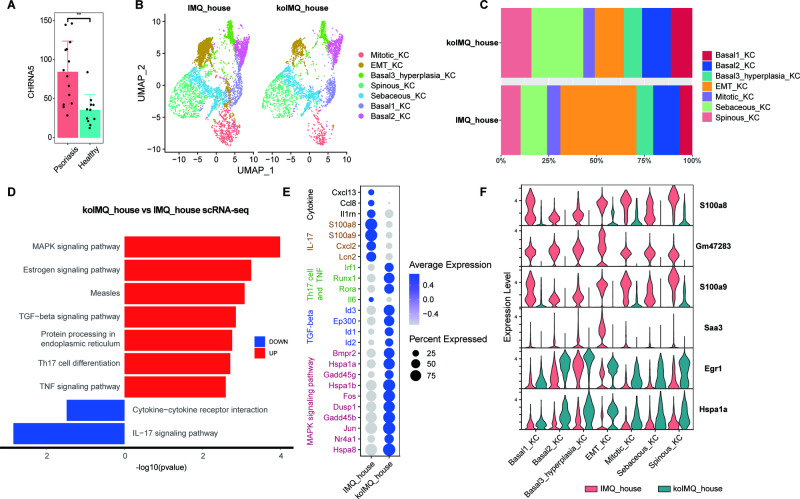
*Chrna*5 KO reduces EMT KCs in psoriasis. **A** Compared to the Healthy group, the expression of *Chrna*5 was increased significantly in the psoriasis group. (GSE171012). **(B)** UMAP plot of keratinocytes in the IMQ house and koIMQ house, coloured by keratinocyte subtype. **C** Proportions of keratinocyte subtypes in the skin of the IMQ group and KO IMQ group. **(D)** KEGG pathway map of koIMQ house vs. IMQ house scRNA-seq. **E** Dot plot of the expression levels of marker genes for different signalling pathways in the IMQ house and koIMQ house. Dot size corresponds to the percent of expressing cells, and dot colour indicates the average expression levels. **F** Violin plot of the expression levels of DEGs in all keratinocyte subtypes. Red represents the IMQ house, and green represents the koIMQ house.

*Chrna5* was shown to be related to cell proliferation, invasion, migration, angiogenesis and immunological functions [25, 26]. Our previous study confirmed that *Chrna5* was overexpressed on the back skin of mice after IMQ treatment. *Chrna5* KO inhibited epidermal proliferation and inflammation in mice. Moreover, scRNA-seq analysis revealed that *Chrna5* KO significantly reduced the number of KCs [23]. We also found high expression of Chrna5 in patients with psoriasis in bulk RNA-seq of clinical samples (Fig. 5A). As shown in Fig. 5B, C, the proportion of EMT KCs was obviously decreased when *Chrna5* was knock out. The KEGG pathway enrichment and marker gene analysis showed that the IL-17 signalling pathway was decreased in the koIMQ house compared to IMQ house (Fig. 5D–F). Combined with the abovementioned results, these data indicate that *Chrna5* KO may reduce EMT KC expression by suppressing the IL-17 signalling pathway.

### Drug verification

**Fig. 6:**
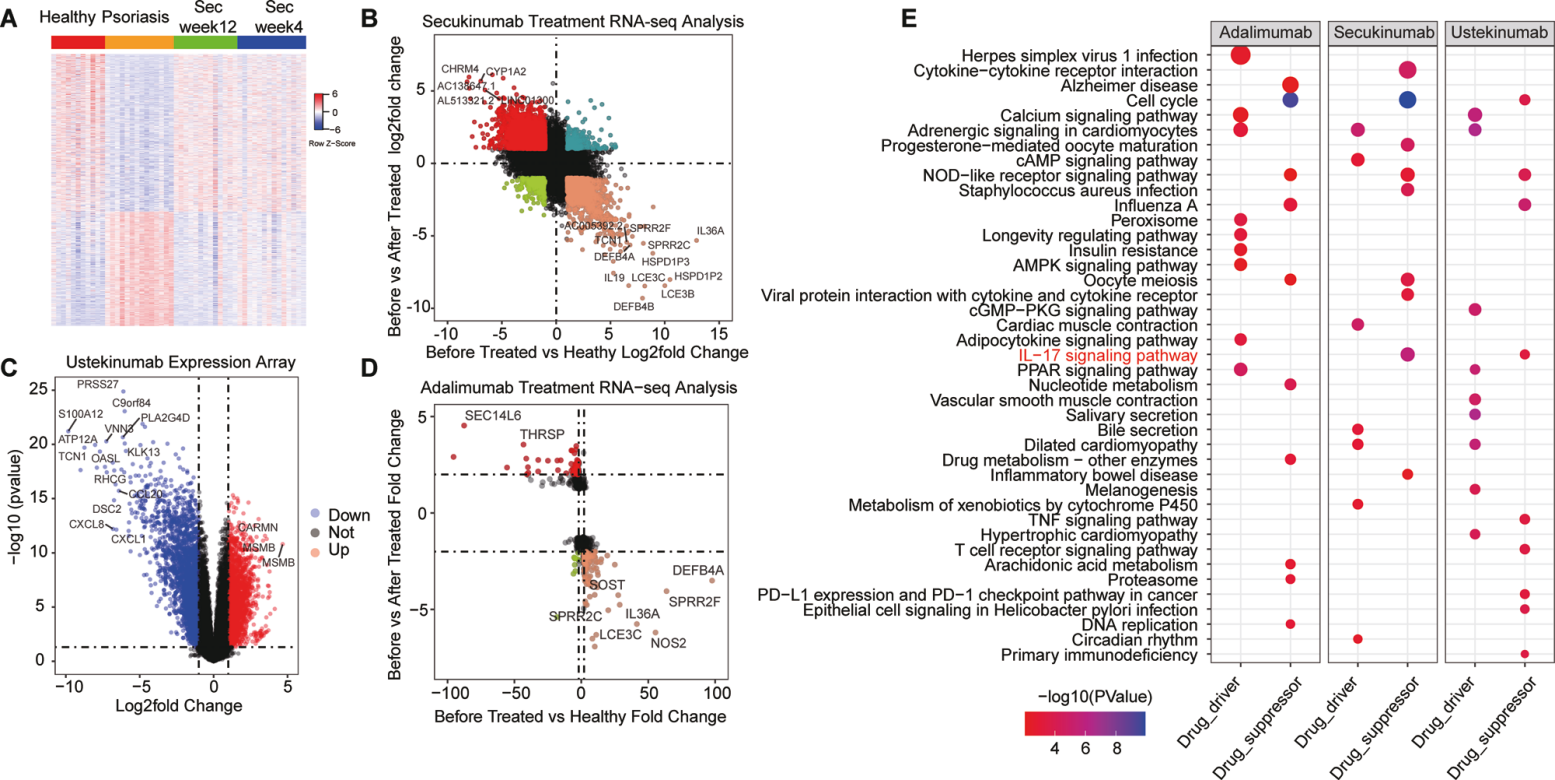
Drug verification. **A** Heatmap of DEGs between the healthy group, psoriasis group, group treated with secukinumab for 12 weeks and group treated with SEC for 4 weeks. (GSE171012). **B** RNA-seq analysis between the healthy group, psoriasis group before treatment and psoriasis group after treatment by SEC. **C** Expression array between the psoriasis group before treatment and psoriasis group after treatment by UST. (GSE103489). **D** RNA-seq analysis between the healthy group, psoriasis group before treatment and psoriasis group after treatment with ADA. (GSE74697). **(E)** Dot plot of the signalling pathways affected by SEC, UST and ADA.

According to the British Association of Dermatologists Guidelines for Biologic Therapy for Psoriasis 2020, three commonly used biological agents were selected: the IL-17A inhibitor SEC, IL-12/23 inhibitor UST and TNFα inhibitor ADA. To determine the related genes affected by each biological agent, we performed RNA-seq analysis and expression array analysis (Fig. 6A–D). If the gene expression in psoriasis is downregulated compared with that in healthy individuals but upregulated when a drug is applied, this kind of genes are called drug driver genes. Otherwise, they’re called drug suppressor genes. For UST, due to the lack of healthy tissue, we called the downregulated genes drug suppressor genes when the drug was applied compared with that in psoriasis. Otherwise, we called them drug driver genes. With these upregulated and downregulated genes, associated signalling pathways were enriched (Fig. 6E). UST and SEC were shown to be closely related to the IL-17 signalling pathway, which was consistent with our scRNA-seq analysis.

To validate the results, we scored the genes suppressed by three biological agents in a single-cell subpopulation. A UMAP plot of IL-17 and its receptor expression showed that IL-17ra was mainly expressed in FBs and that IL-17rc was mainly expressed in KCs (Fig. S5A). The KC subtype with a high SEC score was consistent with that highly expressing IL-17rc (Fig. S5B, C). Similarly, the FB subtype with a high ADA score, SEC sore and UST score was consistent with that with high IL-17ra expression (Fig. S5D–F). As shown in Fig. 3A, the FB subtypes were iFib1 and iFib2, which indicated that iFib subtypes were the main subtypes affected by these biological agents.

### Correlation analysis between drug intervention and EMT

**Fig. 7:**
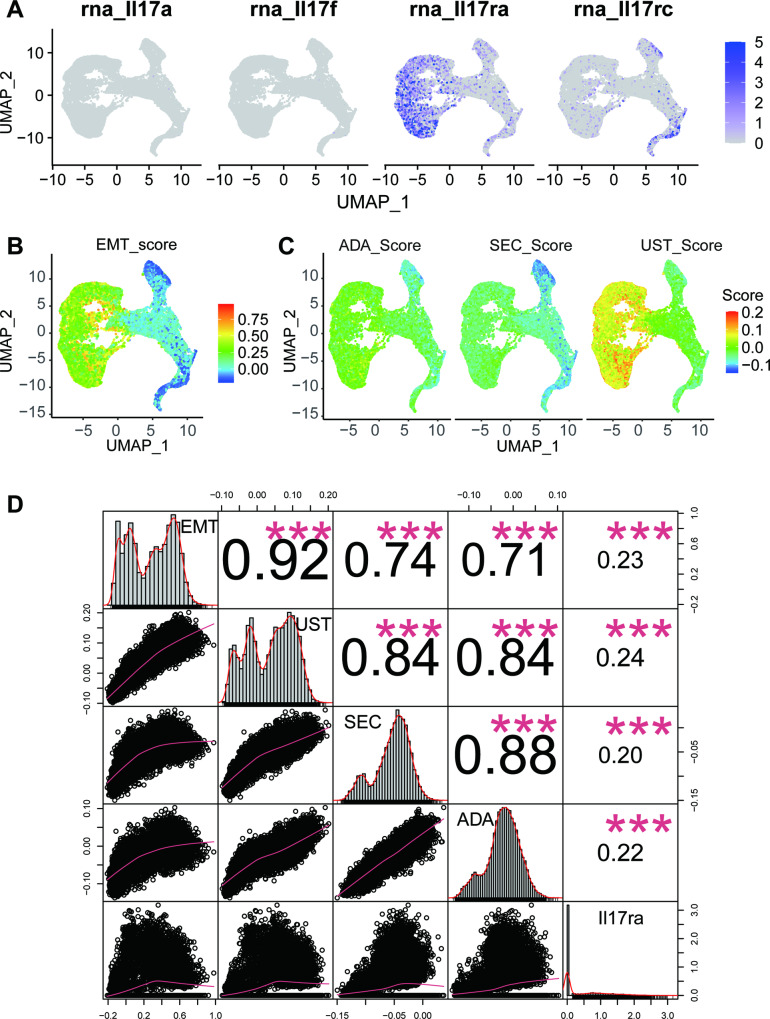
Correlation analysis between drug intervention and EMT. **A** UMAP plot of IL-17 and its receptor expression in EMT-associated cells. **B** UMAP plot of EMT score in EMT-associated cells. **C** UMAP plot showing the distribution of genes upregulated by ADA, SEC and UST in EMT-associated cells. Each dot denotes a single cell; the higher the cell score, the better the effect of the drug. **D** Correlation diagram of EMT and drug intervention.

To determine why EMT subpopulations are affected by the IL-17 signalling pathway, we first detected the expression levels of different IL-17 receptors in EMT subpopulations. We found that iFib was the main cell subpopulation expressing Il17ra (Fig. 7A), with the highest EMT score (Fig. 7B). The genes upregulated by the three biological agents were first scored in EMT-associated cells (Fig. 7C). Obviously, IL-17ra was mainly expressed in iFibs, again indicating that iFib subtypes were the main subtype affected by these biological agents. As shown by the visualized score, both the EMT score and drug intervention score are high in iFib subpopulations. To further strengthen the correlation, we performed correlation analysis (Fig. 7D). The relative coefficients of the EMT score vs. the three drug scores were 0.92, 0.74, and 0.71, which indicated that EMT was highly positively correlated with drug intervention.

### Cell communication

**Fig. 8:**
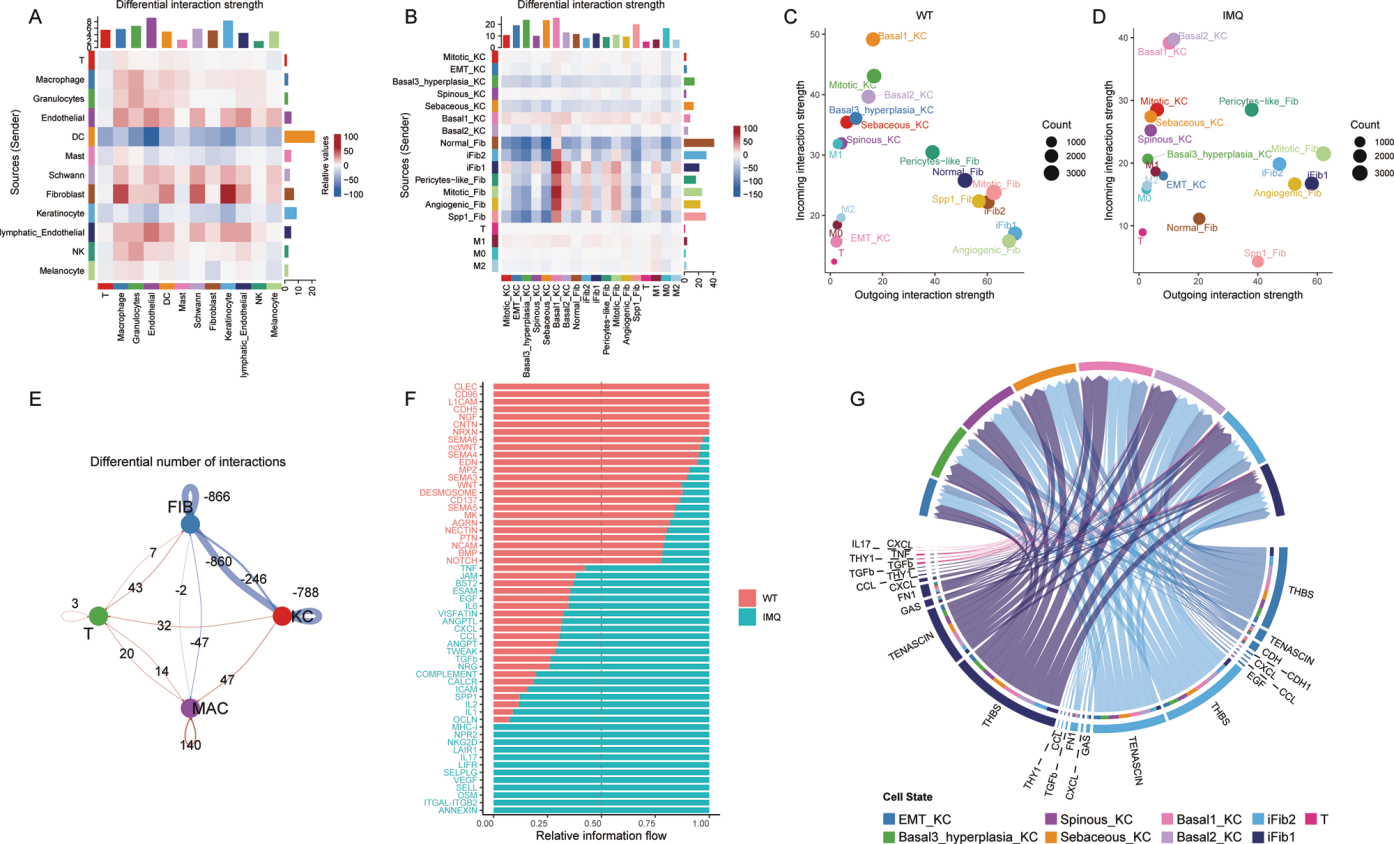
Cell communication. **A** Total interaction strength or weights between different cell type in WT and IMQ group. **B** Total interaction strength or weights between KCs, FBs, T and macrophage subtypes. Scatter plot showed the dominant outgoing and incoming interaction strength in WT (**C**) and IMQ (**D**) group. Incoming is the receiver signalling and outgoing is the sender signalling. **E** Differential number of interactions between IMQ and WT groups in KCs, FBs, T and macrophage cell type. Red indicates that the frequency of interaction strength in the IMQ group increases, and blue indicates that it weakens. **F** Signalling pathway differences in cell communication between WT and IMQ group in KCs, FBs, T and macrophage cell type. **G** Circle plot shows signalling pathways of important communications between T, iFib and KCs in IMQ group.

Cellchat was used to analyze cell communication between cell populations and subpopulations. We found that the intensity of cell communication between Fibs and KCs was strongest among cell populations (Fig. 8A). The cell communication between Fibs, KCs, T and macrophages showed that Fibs communicated strongly with Basal KC cells (Fig. 8B). Comparing the communication between cell subpopulations in the IMQ and WT group, although the overall communication intensity became weaker in the IMQ group, the weight of iFib and EMT KC receiving and releasing communication signals became higher (Fig. 8C, D). Interestingly, Fibs and Basal KC interact with T cells more frequently, and signalling pathways such as IL17, TGFb, and TNF were significantly activated in the IMQ group (Fig. 8E, F). The communication of EMT KC, iFib and T cell subpopulations with KCs and iFib cell subpopulations showed that IL17, TGFb and TNF can promote cell communication between these cell subpopulations (Fig. 8G). Cellular communication can occur between KCs and iFib through extracellular matrix signals such as THBS, CDH, and CXCL (Fig. 8G), which are factors affecting EMT.

### IL-17 signalling pathway verification

**Fig. 9:**
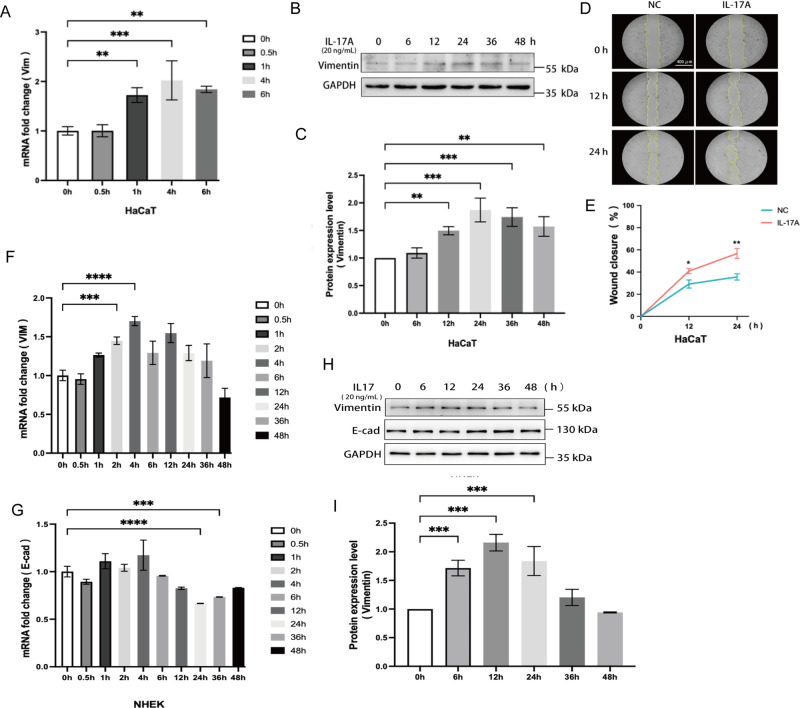
The role of IL-17 in the EMT phenotype. **A** Quantification of *Vim* mRNA expressions in HaCaT cells stimulated with IL-17 (20 ng/mL) for indicated time. **B** Western blot image of Vim expressions in HaCaT cells treated with IL-17 for indicated time. **C** Histogram of protein expression level in **B**. **D** Wound healing assay to show the migration ability of HaCaT cells stimulated with IL-17 (magnification: 10×, Bar = 400 μm). **E** Line graph of the percentage of migration area in **G**. *P < 0.05, **P < 0.01. NC, negative control. **F** Quantification of *Vim* mRNA expressions in NHEK cells stimulated with IL-17 (20 ng/mL) for indicated time. **G** Quantification of *E-Cad* mRNA expressions in NHEK cells stimulated with IL-17 (20 ng/mL) for indicated time. **(H)** Western blot image of Vim expressions in NHEK cells treated with IL-17 for indicated time. **I** Histogram of protein expression level in **B**.

To confirm whether IL-17 signalling is directly involved in the EMT phenotype of the IMQ-induced psoriasis model, we conducted in vitro experiments to verify the role of IL-17. First, we treated the human keratinocyte line HaCaT with IL-17 (20 ng/ml) and detected the expression of EMT-related genes, *Vimentin (Vim)*, by using qRT‒PCR. The results showed that IL-17 treatment upregulated the mRNA expression of *Vim* (Fig. 9A), which is consistent with the results of single-cell sequencing. The upregulation of mRNA occurs within one hour, indicating that this effect is directly caused by IL-17. The WB experiment further confirmed our results (Fig. 9B, C). The results showed that after 12 h of IL-17 treatment, there was a significant upregulation of Vim protein expression, which could be maintained for 48 h. We further conducted a wound healing assay and found that IL-17 can induce the migration of HaCaT cells(Fig. 9D, E). To further confirm whether the effect of IL-17 also exists in human cells, we repeated this experiment using primary cultured human keratinocytes (NHEK). As shown in Fig. 9F, we treated NHEK with IL-17 (20 ng/ml) and detected the expression of *Vim*, by using qRT‒PCR. The results showed that IL-17 treatment upregulated the mRNA expression of *Vim* in 2 h. The expression of *E-cad* mRNA decreased after 24 h, indicating that the weakening of epithelial phenotype may be a more downstream event (Fig. 9G). The WB results further confirmed our results, and a significant increase in Vim protein was detected after 6 h of IL17 treatment (Fig. 9H, I). Therefore, our results suggest that IL-17 is the main inducer of the EMT process in the occurrence of psoriasis.

## Discussion

Psoriasis has long been a heavy burden for patients worldwide because of its repeated attacks and prolonged skin damage, accompanied by itching. Although biologic treatments for psoriasis have achieved excellent results, due to the high cost, this treatment is not affordable for everyone. Therefore, it is urgent to further study the pathogenesis of psoriasis in depth and provide new ideas for more popular treatments. In this study, 13 samples were collected from the database published thus far, and scRNA-seq was performed to construct a single-cell transcriptome map of psoriasis.

As an autoimmune disease, various cells, including KCs, FBs, pDCs, NKT cells, and macrophages, are involved in the entire immune process of psoriasis [3, 4]. Jin et al. used scRNA-seq to reveal the immune cell spectrum of IMQ-induced mice with psoriasis and observed that the proportions of macrophages, NK/T cells, cDCs and pDCs were increased in IMQ-treated mice compared with WT mice [27]. In our scRNA-seq analysis, the proportion of KCs and FBs showed the most significant difference, which indicated that KCs and FBs played important roles in the pathogenesis of psoriasis.

KCs are not only important cytokine-producing cells but also important cytokine target cells and important immune cells [28]. Interestingly, in the process of interpreting the heterogeneity of KCs, we discovered that a subtype of EMT KCs increased obviously in the IMQ group compared with the WT group. Moreover, knocking out Chrna5 significantly reduced the number of KCs in psoriasis, and EMT KCs were the main population that was obviously decreased. This finding indicated that the EMT phenomenon might play an essential role in the development of psoriasis. Fibroblast growth factor 2 was shown to accelerate the EMT process in KCs during the wound healing process [29]. Yan Shi et al. revealed that ROS can promote hypoxia-induced KC EMT by inducing SOX2 expression and activating Wnt/β-catenin [30]. The role of EMT in psoriasis needs to be explored further. As a relatively typical keratinocyte, spinous KCs are another subpopulation that mainly influences psoriasis. These cells can express the IL17rc gene and are one of the main subpopulations that is influenced by interleukin therapy [31]. However, the exact role of spinous KCs in psoriasis remains unclear. More research is needed.

Then, in the mapping and identification of FBs, iFibs exhibited special heterogeneity in psoriasis. Compared with that of the WT group, the proportion of 2 iFib subpopulations increased clearly in the IMQ group. Combined bulk RNA-seq and scRNA-seq analysis also confirmed this result, indicating the important role of iFibs in psoriasis. Current studies have noted that FBs in patients with psoriasis can maintain the hyperproliferative state of the psoriatic epidermis and produce higher levels keratinocyte growth factor [32, 33]. These results suggested that the high level of cytokines released by psoriatic FBs may be an important initiating factor for the excessive proliferation of KCs [34]. Ma et al. reported that using a combination of single-cell and spatial RNA sequencing, they identified an SFRP2^+^ fibroblast subset, which can directly and indirectly augment IL-17A and TNF inflammatory responses. These researchers also described FB-KC crosstalk in amplifying inflammatory responses in psoriatic skin involving IL-17/IL-36 responses, which is consistent with our results [35]

More interestingly, iFibs also had a high EMT score in our scRNA-seq analysis, which indicated that KCs and FBs may interact with each other via EMT. EMT is a biological process by which epithelial cells lose polarity and adhesion and acquire the migratory and invasive properties of mesenchymal cells [36]. This process plays a key role in the regulation of tumour invasion and metastasis in many human cancers [37–39]. During wound healing, tissue regeneration, and fibrosis, EMT is triggered by inflammation [40–42]. Our findings revealed that EMT truly occurs in psoriasis and may be involved in its pathogenesis. The EMT process in psoriasis was a dynamic process between EMT KCs and iFibs. To confirm the EMT process in psoriasis, we conducted drug verification, correlation analysis and cell communication. The results indicated that EMT may play an essential role in the pathogenesis of psoriasis, which needs further investigation.

The process of EMT is regulated by an aggregation of signalling pathways and signalling molecules in different contexts. However, the signalling pathway regulating the EMT process during psoriasis remains unknown. Harirchian et al. used scRNA-seq analysis to show that aberrant inflammatory transcription of A20 in KCs in psoriasis is related to the IL-17 and TNF-a signalling pathways [43]. In our whole scRNA-seq analysis, several lines of evidence indicate that the IL-17 signalling pathway may be involved in the EMT process. In the analysis of the heterogeneity of KCs and FBs, IL-17 signalling pathway-related genes and KEGG pathway map both showed the close relationship of IL-17 signalling pathway. In the dynamic evolution process of EMT in psoriasis, as the KC marker *Krt5* increased, the EMT markers *Vim* and *Zeb2*, and the IL-17 signalling pathway marker *Il17ra* clearly decrease. Additionally, we performed in vitro experiments using qRT‒PCR, WB and wound healing assays, which confirmed that IL-17 is the inducer of the EMT process in the occurrence of psoriasis. Taken together, the IL-17 signalling pathway may be the pathway with the most potential to participate in the EMT process of psoriasis.

Collectively, based on an unbiased single-cell RNA-seq analysis, we presented the full-cycle landscape of key cells associated with psoriasis, such as KCs and FBs. Most interestingly, we revealed that EMT KCs, iFibs and their crosstalk through EMT may play a critical role in the pathogenesis of psoriasis, in which the IL-17 signalling pathway may be the most likely pathway involved.

## Materials and Methods

### Data collection, code availability and grouping

Mouse psoriasis scRNA-seq data including all KC or FB populations published before September 2022 were selected. The sequencing data generated in this study are available at the Gene Expression Omnibus (GEO, https://www.ncbi.nlm.nih.gov/geo/), and the Genome Sequence Achieve (GSA, https://bigd.big.ac.cn/gsa/). Related accession numbers were detailed below. Finally, thirteen samples divided into five groups were collected in this study: 4 imiquimod-induced psoriatic mouse samples (IMQ group), 5 wild-type mouse samples (WT group), 2 mouse samples that were induced by imiquimod and treated with SHP099 hydrochloride for an additional 4 days (SHP099 group), 1 Chrna5 knockout mouse sample (KO group), and 1 Chrna5 knockout imiquimod-induced psoriasis sample (KO IMQ group). The data of four mouse samples, 1 WT, 1 KO, 1 IMQ and 1 KO IMQ, were obtained from our previous study (GSM5795802, GSM5795803, GSM5795800 and GSM5795801) [23]. The other 9 samples were extracted from published datasets. GSM4547483 was data from the ears of C57BL/6 mice treated with IMQ for 5 consecutive days. GSM5024748 and GSM5024749 included C57BL/6J mice treated with IMQ on the shaved dorsal skin for 4 consecutive days. GSM5024750 and GSM5024751 were from the IMQ-induced psoriasis mouse model treated with SHP099 hydrochloride for an additional 4 days. GSM4547481 and GSM4547482 included the dermis and epidermis of untreated WT mice, respectively. GSM5024746 and GSM5024747 were data on untreated WT mice. Detailed information on the 13 samples is summarized in Supplementary Table 1. The bulk RNA-seq data used in Fig. 3E were extracted from GEO with the accession number GSM2299980(WT), GSM2299981(WT), GSM229998 (IMQ), GSM2299983 (IMQ). Other data used in Fig. 6 were extracted from GEO with accession numbers GSE171012, GSE103489, and GSE74697.

### RNA-seq analysis

The RNA-seq data were obtained from GEO with accession numbers GSM2299980, GSE171012 and GSE74697. The FastQC tool (http://www.bioinformatics.babraham.ac.uk/projects/fastqc/) was applied to analyse the raw data. Then, Trimmomatic (http://www.usadellab.org/cms/index.php?page=trimmomatic) was deployed to eliminate low-quality sequences and adaptor-contaminated sequences. The filtered data were mapped to mouse mm10 or the human hg19 genome using HISAT2 (https://daehwankimlab.github.io/hisat2/). SAM files were generated and then converted to BAM format with SAMtools [44, 45]. HTseq [46] was employed to calculate the number of reads mapped on each gene. The DEGs were then screened from the mapping results using the R package DEseq2 [47], and those with *P* < 0.05 and log2FoldChange value > 1 or log2FoldChange value <−1 were identified as genes with significant differences.

### Microarray analysis

GEO2R was used to compare samples between the psoriasis group before treatment and the psoriasis group after treatment with UST to identify DEGs.

### ScRNA-seq analysis

The Seurat package (version 4.2.0) was applied to transform the matrix to a Seurat object. Quality filtering was carried out according to the sequencing characteristics. Samples with fewer than 1000 cells were eliminated. The data were standardized by CCA to erase the influence of data sources. Then, the “ScaleData” function was used for further linear regression against the normalized expression levels for each gene, the total UMI counts and mitochondrial RNA content per cell. Subsequently, principal component analysis (PCA) with “RunPCA” was performed. Eventually, gene expression and clustering results were visualized on a UMAP plot of the top ten PCs using “RunUMAP”, and “FindNeighbors” and “FindClusters”, which were used to cluster cells.

### Annotation of cell types

Cell type was determined based on the gene expression of known markers, e.g., Rgs1, Cd3g, Cd3e, and Cd69 for T cells; Plp1, Mpz, Gatm, Mbp, and Mal for Schwann cells; Gzmb, Klrd1, Ptprcap, Xcl1, and Kirk1 for NK cells; Dct, Tyrp1, Kit, Ptgds, and Pax3 for melanocytes; Hdc, Ccl6, Srgn, Ccl3, and Lilr4b for mast cells; Lyz2, Fcer1g, and Ctss for macrophages; Ccl21a, Mmrn1, Cldn5, and Lvve1 for lymphatic endothelial cells; Krt5, Krt14, Lgals7, Krt17, and Sfn for keratinocytes; Retnlg, Wfdc21, Wfdc17, and Cxcl2 for granulocytes; Dcn, Col3a1, Col1a2, Col1a1, and Clo6a3 for fibroblasts; Ptptb, Fit1, Vwf, and Podxl for endothelial cells; H2-Ab1, H2-Eb1, H2-Aa, and H2-DMa for DCs; and Cfd, Car3, Cidec, and Adig for adipocytes. Annotation of the subcell types of KCs and FBs is based on the combination of known marker expression and participating signalling pathways.

### Signature scores

Based on 194 EMT genes reported by Yutong Sha et al. [48], the “AddModuleScore” (Seurat, version 4.2.0) function was employed to calculate the EMT signature score of all clusters. By estimating EMT meta-signature scores between different groups based on the nonparametric Wilcoxon rank sum test and determining the average expression level of the 194 genes for each cell, we obtained the EMT signature score. The human genes inhibited by the three drugs were converted into mouse genes through orthologous methods and used to score EMT-related subcelltype in scRNA-seq.

### Gene set variation analysis

To explore the function of different KC subtypes and FB subtypes, we estimated pathway activity through gene set variation analysis (GSVA, version 1.30.0) based on hallmark gene sets from the Molecular Signatures Database (MSigDB, version 6.2)

### Trajectory inference

Trajectory analysis was performed via the R package Monocle (version 2.22.0) to explore the dynamic EMT process between KCs and FBs. The top highly variable genes (HVGs) were differential gene intersections selected from single-cell sequencing and RNA-Seq. Then, the “estimateSizeFactors” and “estimateDispersions” functions were used to build statistical models to characterize the data. Additionally, the “reduceDimension” function was applied to reduce dimensions, and the “orderCells” function was used to place cells onto a pseudotime trajectory.

### Correlation analysis

The R package PerformanceAnalytics was used to determine the correlation between EMT and the three drug interventions. Normalized II17ra expression from Seurat was used as the input data. The “AddModuleScore” (Seurat, version 4.2.0) function was used to evaluate the average expression value of the drug-induced gene set.

### KEGG

KEGG pathway analyses were performed using the DAVID Functional Annotation Bioinformatics Microarray Analysis tool (http://david.abcc.ncifcrf.gov/).

### Analysis of intercellular communication

Intercellular communication networks from scRNA-seq data can be quantitatively inferred, analyzed, and visualized using CellChat [49]. The gene expression data of different cell types identified were input into CellChat, and the CellChatDB.mouse file was used as a reference to generate a network map of the number and intensity of interactions between cells.

### Fluorescent immunohistochemistry

After deparaffinization, slides were hydrated in alcohol. Antigen epitope retrieval was induced by microwave heating. Normal goat serum was used for blocking. For analysis of the expression pattern of candidate antibodies in IMQ and WT tissues, sections were immunostained with primary antibodies overnight at 4 °C, and the secondary antibody used for immunostaining was CoraLite488-conjugated anti-rabbit or CoraLite594-conjugated anti-mouse immunoglobulin (Wuhan Sanying, Proteintech), following the protocol of the manufacturer. DAPI was used for counterstaining. The antibodies are listed in Supplementary Table 2.

### Cell isolation and culture

Skin specimens of young children’s foreskins were obtained from Shandong Provincial Hospital Affiliated to Shandong First Medical University. The study was approved by the hospital’s ethics committee. Informed consent was obtained from all patients. The specimens were digested with dispase II (#BCBR9297V, Sigma-Aldrich, U.S.A) at 4 °C overnight to separate the epidermis from the dermis. Then the epidermal specimens were digested with a 0.25% trypsin (37 °C, 10 min) to obtain single-cell suspensions. NHEKs were grown and maintained in keratinocyte medium (#100-0500, STEM CELL, Canada).

The dermal tissue which cut into 1 mm × 1 mm spices was digested with 5% Collagenase Type I (#2810679, Gibco, U.S.A) to obtain single-cell suspensions in 37 °C incubator. Normal human dermal fibroblast (NHDF) was cultured and expanded in Fibroblast Basal Medium (PCS-201-030, ATCC, U.S.A) supplemented with Fibroblast Growth Kit-Low serum (PCS-201-041, ATCC, U.S.A) and 1% penicillin-streptomycin.

Human keratinocytes (HaCaT cells; GCC-AO0003RT, Genechem, Shanghai, China) were cultured in Dulbecco’s modified Eagle’s medium with 10% FBS and 1% penicillin‒streptomycin. The HaCaT cells were authenticated every three months by STR profiling. Cells were stimulated with recombinant human IL-17A (MCE, HY-P70753, Ann Arbor, MI) in serum-free medium.

### Western blotting

Protein from mouse skin tissues and cells was extracted using RIPA lysis buffer (Strong) (CW2333, CWBIO, Beijing, China), and a high-speed tissue grinder (KZ-II, Servicebio, Wuhan, China) was used to grind the sample for subsequent protein extraction. The extracted protein was separated by SDS‒PAGE and transferred onto a 0.22 μm polyvinylidene difluoride membrane. Primary antibodies, including *Vim* (1:1000; ab8069, Abcam, Cambridge, United Kingdom), E-cadherin (1:5000; 60335-1-Ig, ProteinTech) and GAPDH (1:3000; 60004-1-Ig, ProteinTech), were applied. The primary antibodies and membranes were coincubated at 4 °C overnight. After incubation with the corresponding secondary antibody (mouse [SA00001-1, ProteinTech]), the supersensitive chromogenic reagent (PK10003, ProteinTech) was used for the colour reaction.

### qRT‒PCR

Total RNA was separated with TRIzol reagent (CW0580, CWBIO) and extracted using an RNA simple Total RNA Kit (CW0581S, CWBIO), and cDNA was synthesized using a rapid reverse transcription kit (CW2569M, CWBIO). qRT‒PCR was carried out in a real-time PCR instrument using a SuperReal PreMix Plus (SYBR Green) kit (CW0957M, CWBIO). With GAPDH as the reference gene, the relative expression of the target gene was calculated using the Livak method. The primer sequences are provided in Supplementary Table 3.

### Wound healing assay

HaCaT cells were cultured in a 6-well plate until 100% confluence, and the monolayer was scratched longitudinally and transversely using a 10 μL pipette tip. After the dislodged cells were rinsed away, serum-free medium and its counterpart with IL-17A was added to the wells. The wound area was observed at 0, 12, and 24 h to monitor healing. The images were processed using ImageJ.

### Statistical analysis

Excel and GraphPad Prism (version 9.0) were used for statistical analysis of quantitative data. The data were analysed by two-sided Student’s *t*-test or the Mann‒Whitney test for two groups and by one-way analysis of variance (ANOVA) followed by Bonferroni/Dunn post hoc comparison of means with correction for multiple comparisons. The variance was similar between the groups that were being statistically compared. In all cases, *p* < 0.05 was considered significant.

### Supplementary information

supplemental material

Raw images of western blot

aj-checklist

## Data Availability

The sequencing data generated in this study are available at the Gene Expression Omnibus (GEO, https://www.ncbi.nlm.nih.gov/geo/), and the Genome Sequence Achieve (GSA, https://bigd.big.ac.cn/gsa/). Detailed ID information was shown in Supplementary table 1.
