# Supplementary material for: Single-cell RNA-seq reveals keratinocyte and fibroblast heterogeneity and their crosstalk via epithelial-mesenchymal transition in psoriasis
Source: Cell Death Dis. 2024 Mar 12;15(3):207. doi: 10.1038/s41419-024-06583-z (PMC10933286; doi:10.1038/s41419-024-06583-z)
Supplement: Supplementary file 1 — supplemental material [file 41419_2024_6583_MOESM1_ESM.pdf]

Figure S1

A

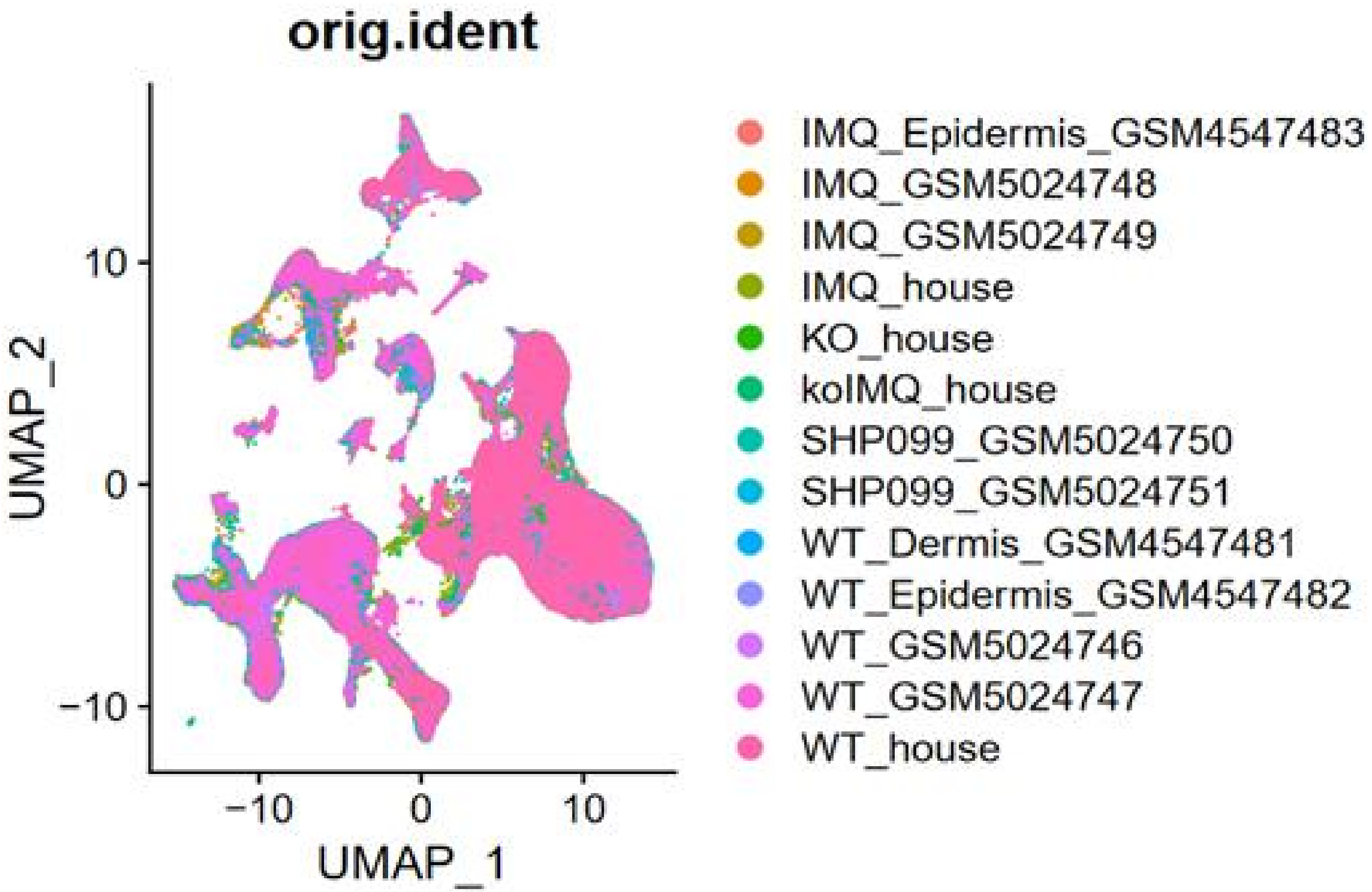
orig.ident
UMAP_2
UMAP_1
10
0
−10
−10
0
10
IMQ_Epidermis_GSM4547483
IMQ_GSM5024748
IMQ_GSM5024749
IMQ_house
KO_house
koIMQ_house
SHP099_GSM5024750
SHP099_GSM5024751
WT_Dermis_GSM4547481
WT_Epidermis_GSM4547482
WT_GSM5024746
WT_GSM5024747
WT_house

B

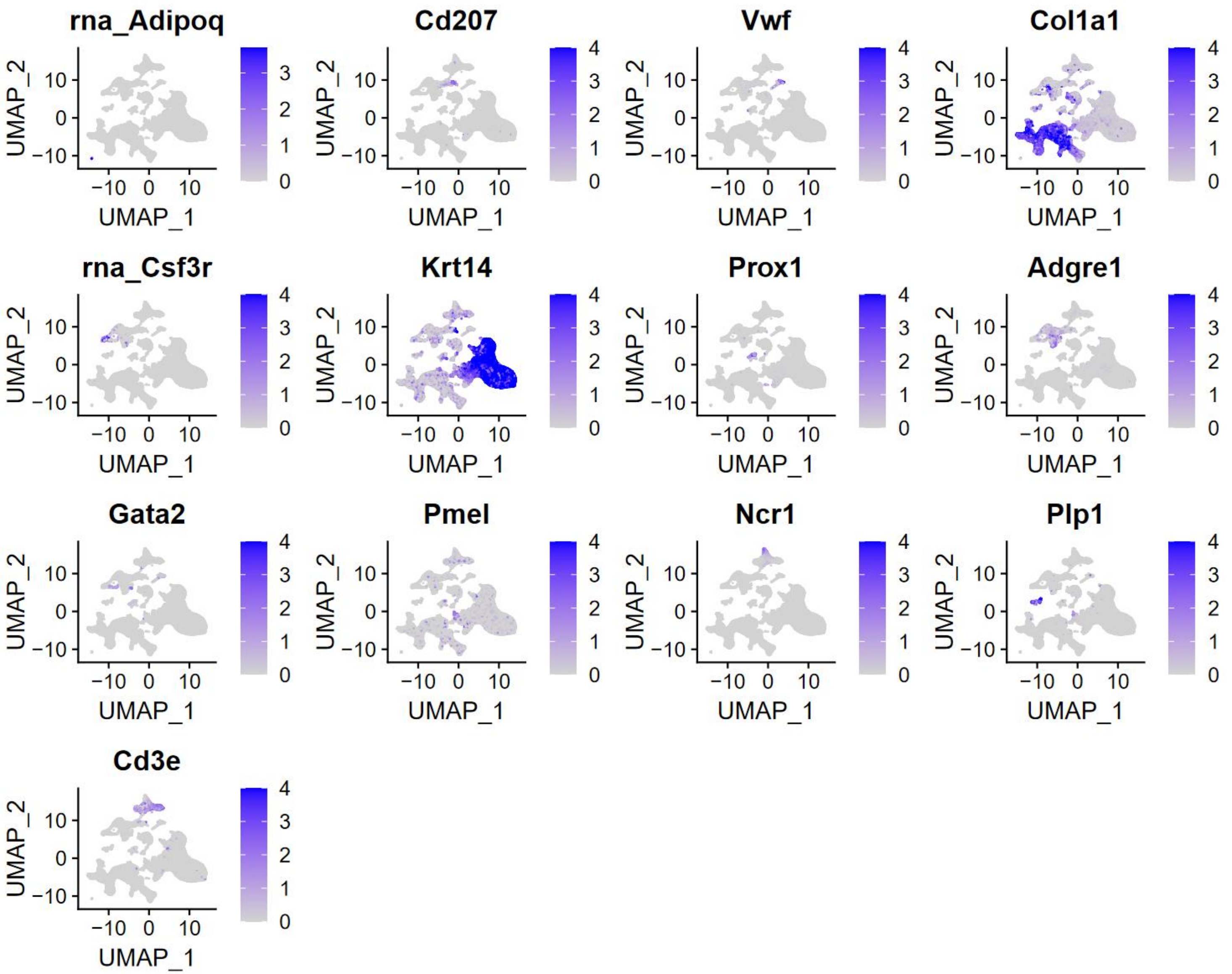
rna_Adipoq
Cd207
Vwf
Col1a1
rna_Csf3r
Krt14
Prox1
Adgre1
Gata2
Pmel
Ncr1
Plp1
Cd3e
UMAP_1
UMAP_2

C

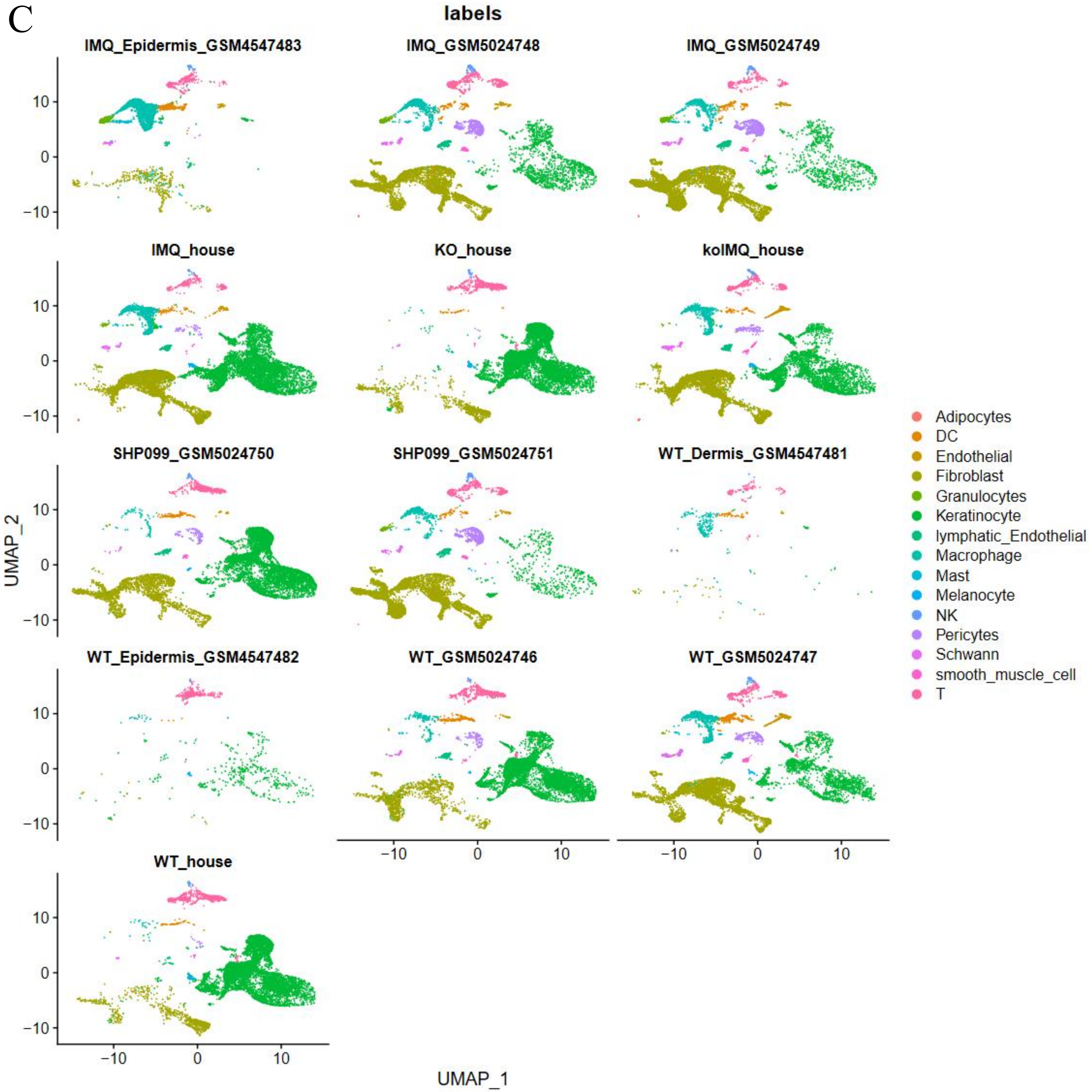
labels
IMQ_Epidermis_GSM4547483
IMQ_GSM5024748
IMQ_GSM5024749
IMQ_house
KO_house
koIMQ_house
SHP099_GSM5024750
SHP099_GSM5024751
WT_Dermis_GSM4547481
WT_Epidermis_GSM4547482
WT_GSM5024746
WT_GSM5024747
WT_house
UMAP_2
UMAP_1
10
0
−10
Adipocytes
DC
Endothelial
Fibroblast
Granulocytes
Keratinocyte
lymphatic_Endothelial
Macrophage
Mast
Melanocyte
NK
Pericytes
Schwann
smooth_muscle_cell
T

FigureS1

(A) UMAP plot of the 94,759 cells from 13 samples in the skin of mice, colored by sample. Each dot denotes a single cell.

(B)UMAP plots illustrating key marker genes to identify cell types as shown in (figure1A),including adipocytes(*Adipoq*), DC(*Cd207*), Endothelial(*Vwf*), Fibroblast(*Col1a1*), Granulocytes(*Csf3r*), Keratinocyte(*Krt14*), lymphatic-Endothelial(*Prox1*), Macrophage(*Adgre1*), Mast(*Gata2*), Melanocyte(*Pmel*), NK(*Ncr1*), Schwann(*Plp1*), T(*Cd3e*).

(C) UMAP plot of the cells from the skin of mice in 13 samples , colored by cell type.

Figure S2

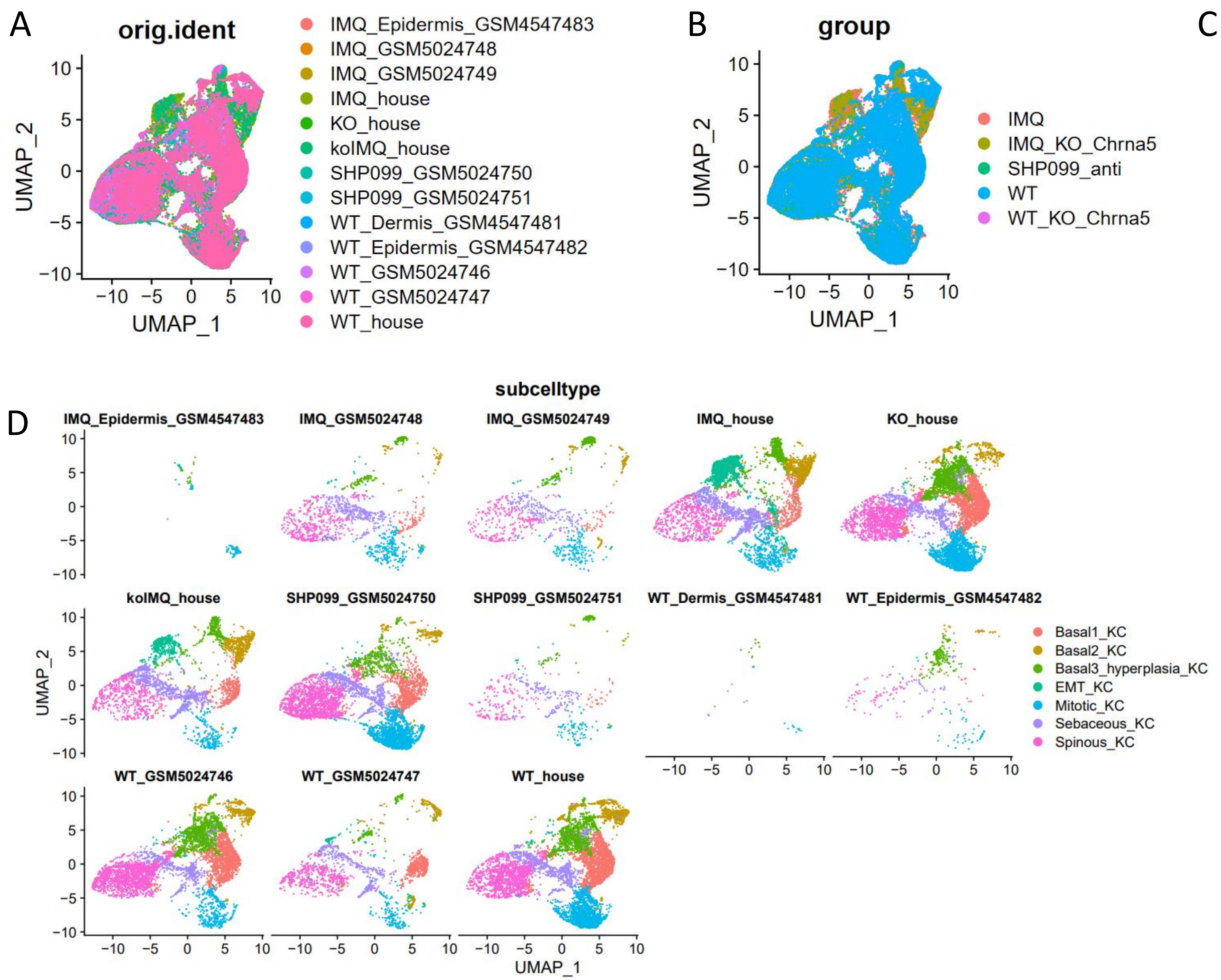

A
orig.ident
IMQ_Epidermis_GSM4547483
IMQ_GSM5024748
IMQ_GSM5024749
IMQ_house
KO_house
koIMQ_house
SHP099_GSM5024750
SHP099_GSM5024751
WT_Dermis_GSM4547481
WT_Epidermis_GSM4547482
WT_GSM5024746
WT_GSM5024747
WT_house
UMAP_1
UMAP_2
B
group
IMQ
IMQ_KO_Chrna5
SHP099_anti
WT
WT_KO_Chrna5
C
EMT_Score
***
Basal1_KC
Basal2_KC
Basal3_hyperplasia_KC
EMT_KC
Mitotic_KC
Sebaceous_KC
Spinous_KC
D
subcelltype
IMQ_Epidermis_GSM4547483
IMQ_GSM5024748
IMQ_GSM5024749
IMQ_house
KO_house
koIMQ_house
SHP099_GSM5024750
SHP099_GSM5024751
WT_Dermis_GSM4547481
WT_Epidermis_GSM4547482
WT_GSM5024746
WT_GSM5024747
WT_house

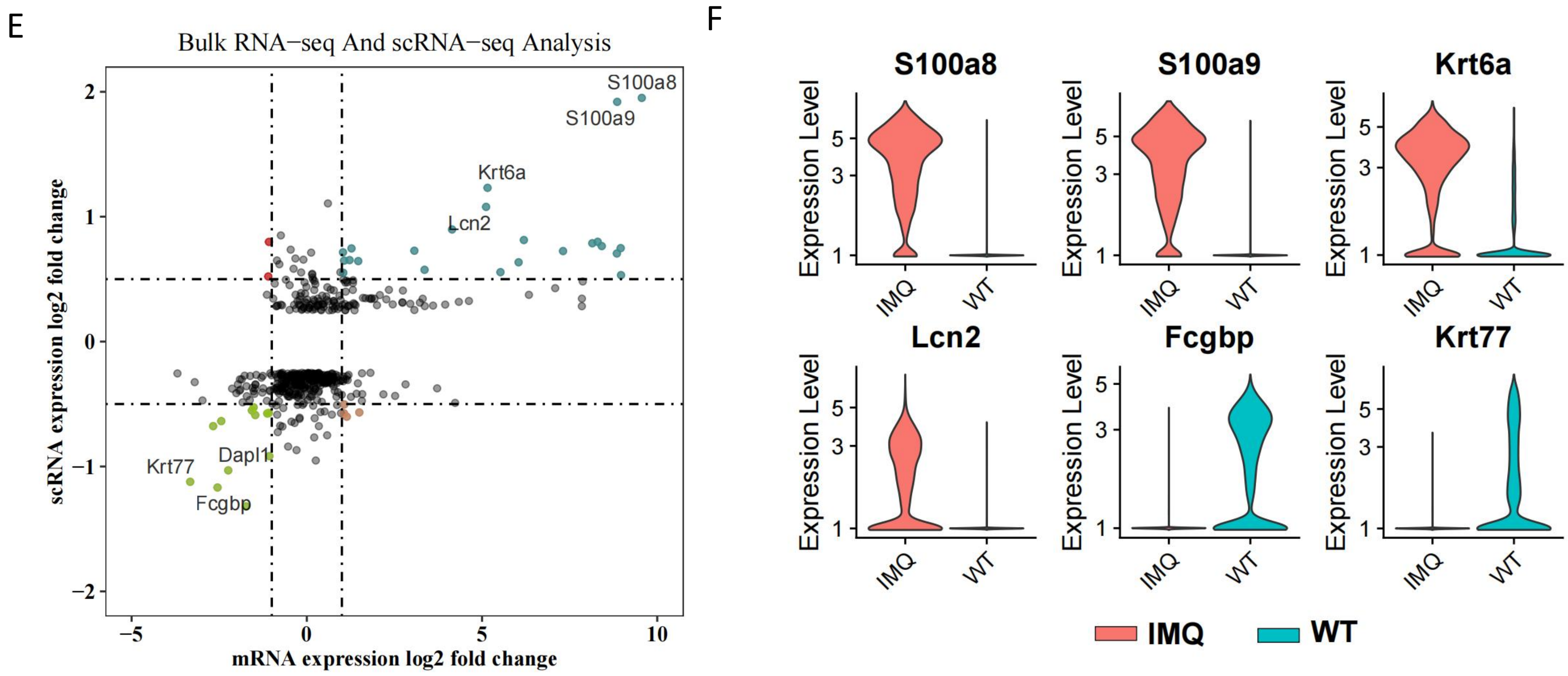
E
Bulk RNA−seq And scRNA−seq Analysis
S100a8
S100a9
Krt6a
Lcn2
Dapl1
Krt77
Fcgbp
scRNA expression log2 fold change
mRNA expression log2 fold change
F
S100a8
S100a9
Krt6a
Lcn2
Fcgbp
Krt77
Expression Level
IMQ
WT

FigureS2

(A,B) UMAP plot of 42073 keratinocyte coloured by sample(A) and group(B). Each dot denotes a single cell.

(C) Box plot of the scores of keratinocyte subtypes. The boxes show lower quartile, middle quartile and upper quartile. Whiskers represent 1.5 times the interquartile range (IQR). P value was calculated by Wilcoxon test (*,p < 0.05;**,p < 0.01;***,p < 0.001).

(D) UMAP of all keratinocyte subtypes from 13 different samples.

(E) Bulk RNA-seq and scRNA-seq analysis of the DEGs of the KCs in the IMQ group and WT group. Bluk RNA-seq data source: IMQ (GSM2299982,GSM2299983) vs WT (GSM2299980,GSM2299981)

(F)Violin plots illustrating the DEGs of the KCs in the IMQ group and WT group.

FigureS3

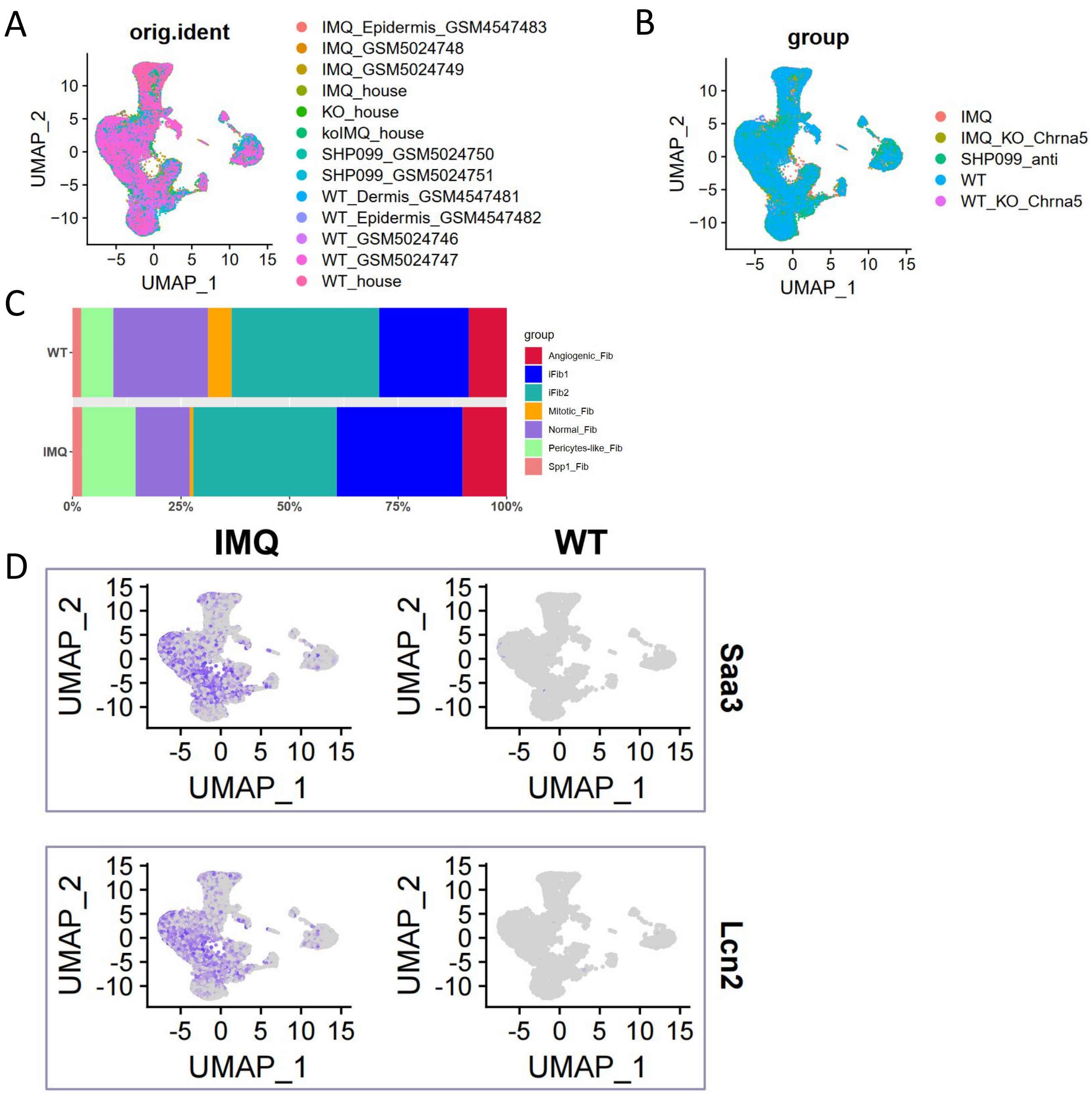
A
orig.ident
IMQ_Epidermis_GSM4547483
IMQ_GSM5024748
IMQ_GSM5024749
IMQ_house
KO_house
koIMQ_house
SHP099_GSM5024750
SHP099_GSM5024751
WT_Dermis_GSM4547481
WT_Epidermis_GSM4547482
WT_GSM5024746
WT_GSM5024747
WT_house
UMAP_1
UMAP_2
B
group
IMQ
IMQ_KO_Chrna5
SHP099_anti
WT
WT_KO_Chrna5
C
WT
IMQ
0%
25%
50%
75%
100%
group
Angiogenic_Fib
iFib1
iFib2
Mitotic_Fib
Normal_Fib
Pericytes-like_Fib
Spp1_Fib
D
IMQ
WT
Saa3
Lcn2

FigureS3

(A,B) UMAP plot of 33118 fibroblast colored by sample(A) and group(B). Each dot denotes a single cell.

(C) Proportions of fibroblast subtypes in the skin of IMQ group and WT group.

(D) UMAP plots illustrating the two key marker genes(Saa3 and Lcn2) which show the biggest expression difference in IMQ and WT group.

FigureS4

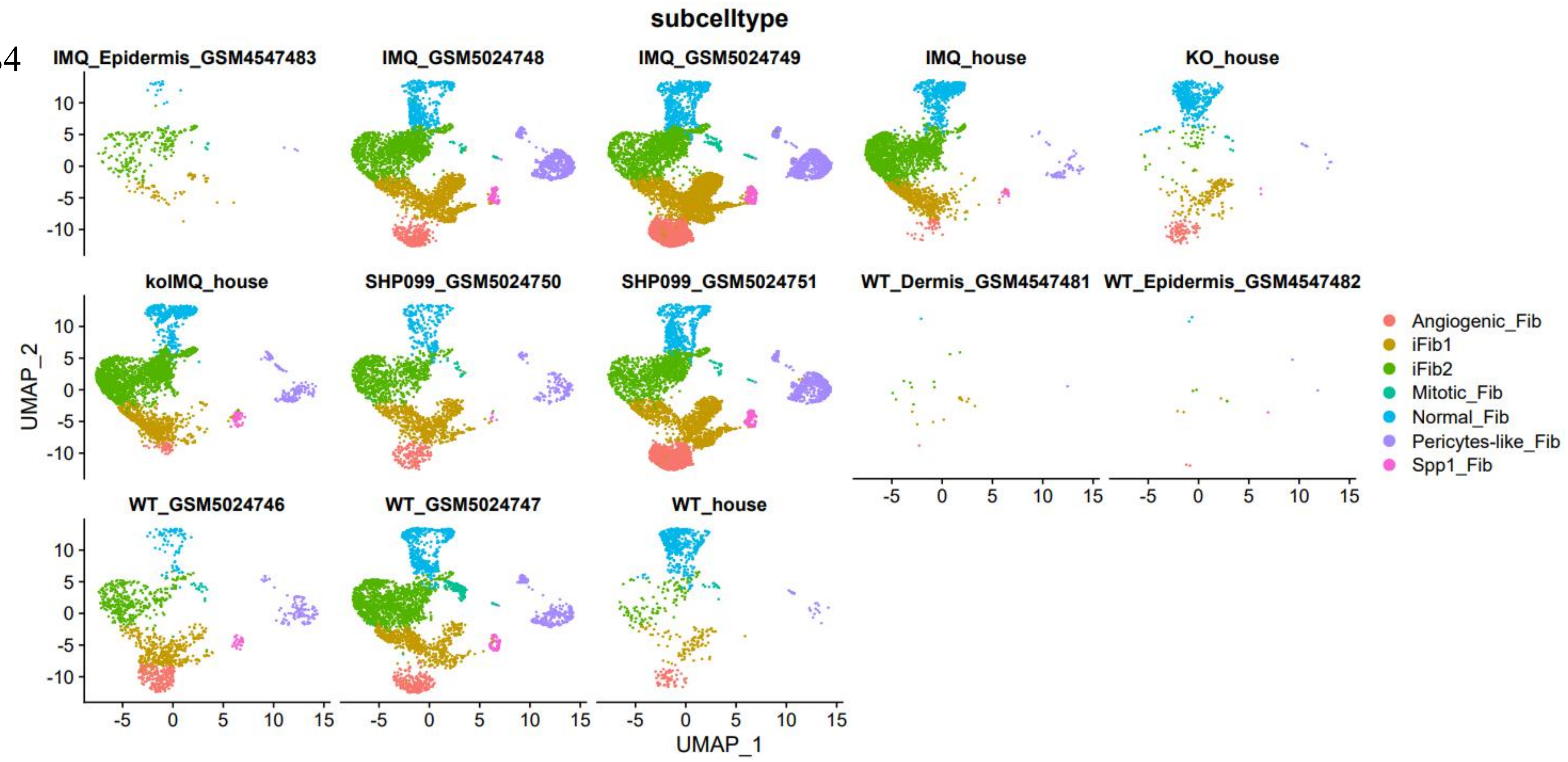

FigureS4 UMAP of all fibroblast subtypes from 13 different samples.

FigureS5

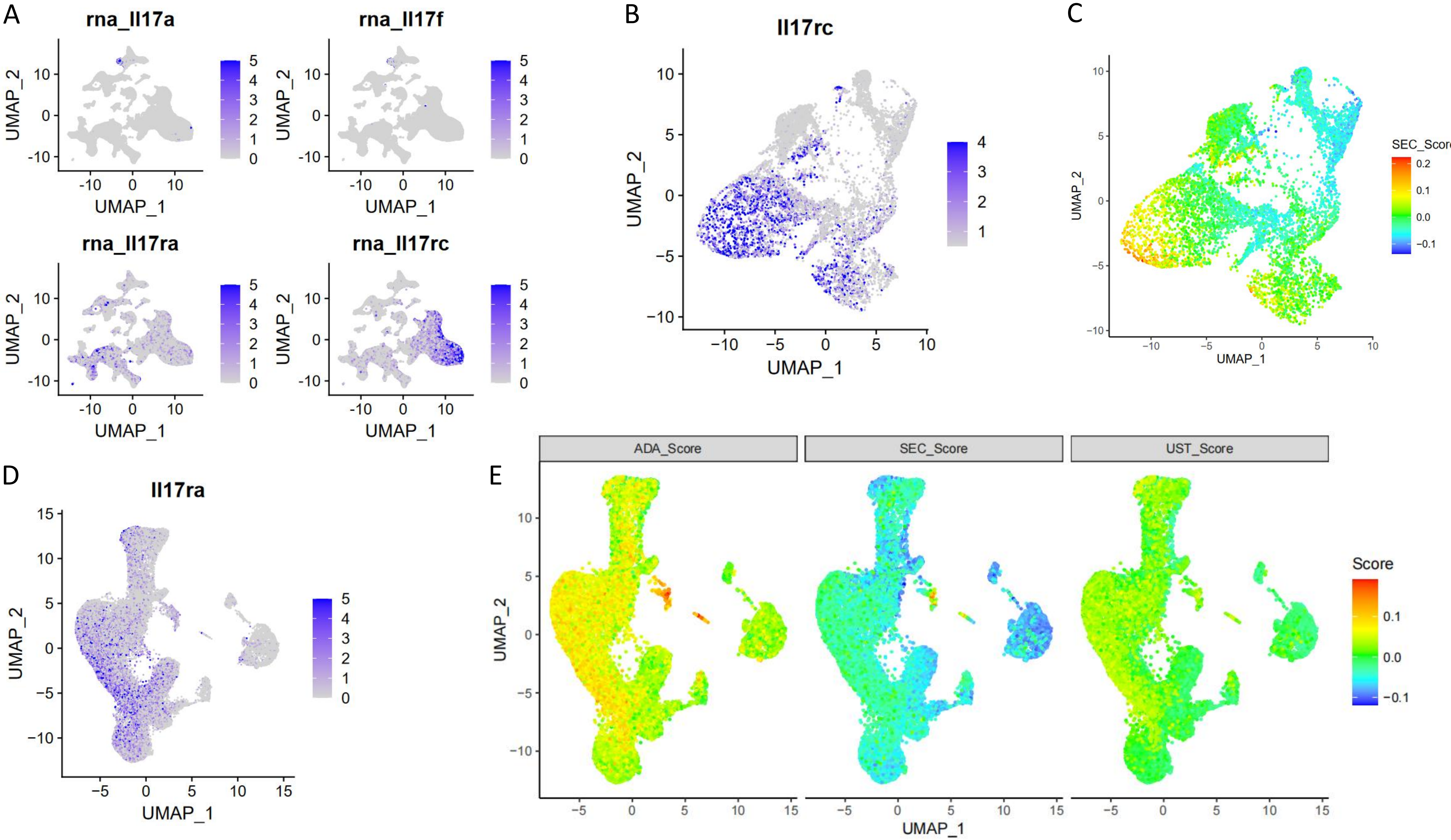
A
rna_Il17a
rna_Il17f
rna_Il17ra
rna_Il17rc
UMAP_1
UMAP_2
B
Il17rc
C
SEC_Score
D
Il17ra
E
ADA_Score
SEC_Score
UST_Score
Score

FigureS5

(A) UMAP plot of IL-17 and its receptor expression in the skin of mice.

(B) UMAP plot of IL-17rc expression in keratinocyte.

(C) UMAP plot showing the distribution of downregulated genes affected by SEC in keratinocyte . Each dot denotes a single cell, the higher the cell score, the better the effect of the drug.

(D) UMAP plot of IL-17ra expression in fibroblast.

(E) UMAP plot showing the distribution of downregulated genes affected by SEC, UST and ADA in fibroblast .

FigureS6

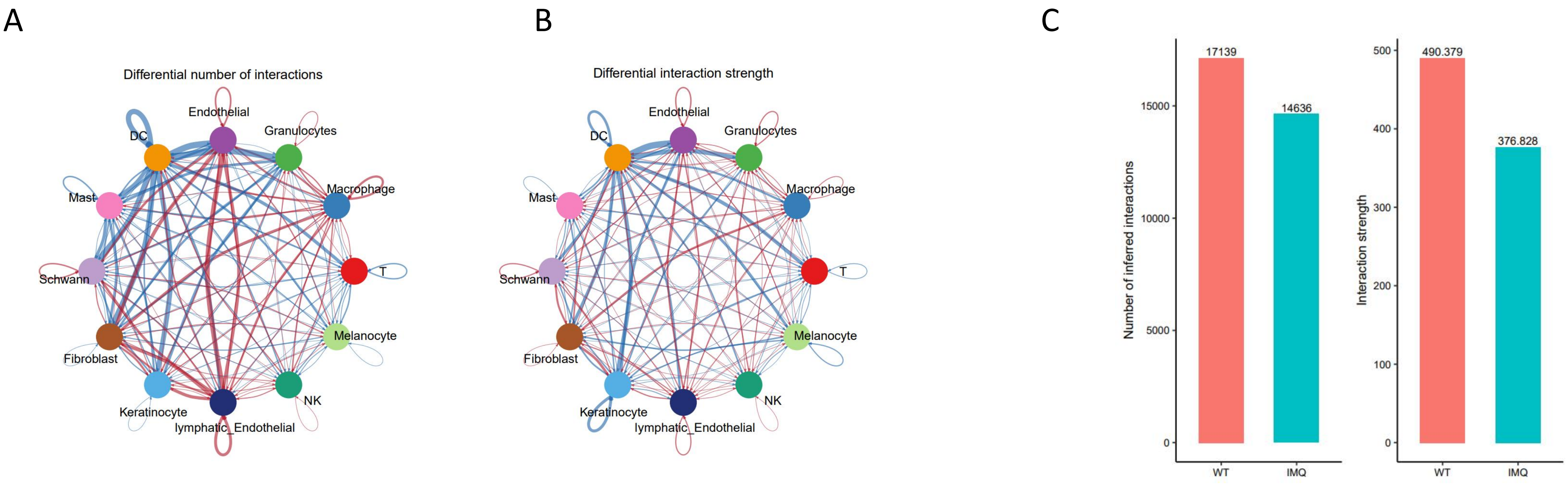

FigureS6

(A-B) Calculate the aggregated cell-cell communication network, the number of interactions(A) and the total interaction strength or weights(B) between any two cell groups were displayed.

(C) the number of interactions and the total interaction strength or weights between IMQ and WT group in in KCs, FBs, T and macrophage cell type.

FigureS7

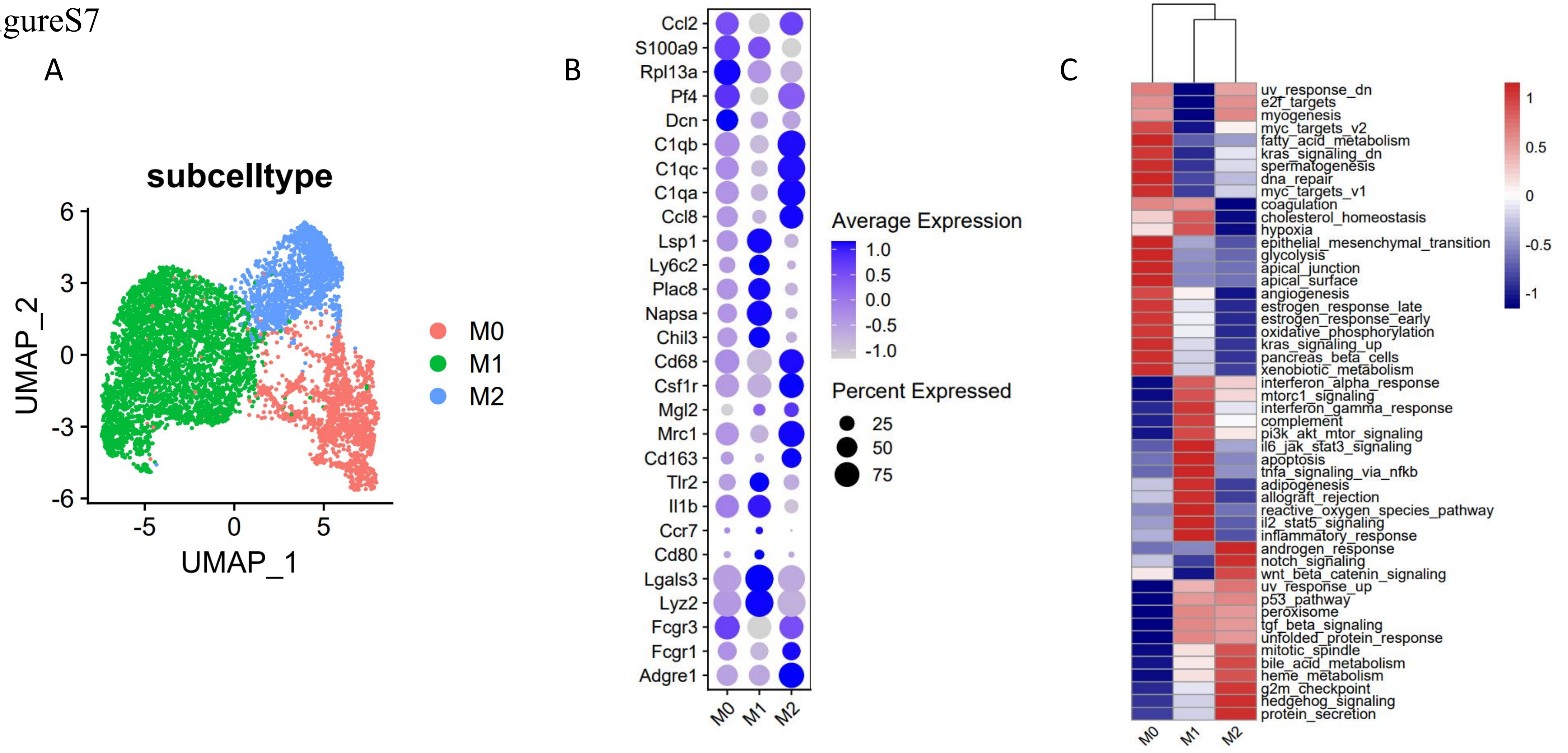
A
subcelltype
UMAP_1
UMAP_2
6
3
0
-3
-6
-5
0
5
M0
M1
M2
B
Ccl2
S100a9
Rpl13a
Pf4
Dcn
C1qb
C1qc
C1qa
Ccl8
Lsp1
Ly6c2
Plac8
Napsa
Chil3
Cd68
Csf1r
Mgl2
Mrc1
Cd163
Tlr2
Il1b
Ccr7
Cd80
Lgals3
Lyz2
Fcgr3
Fcgr1
Adgre1
M0
M1
M2
Average Expression
1.0
0.5
0.0
-0.5
-1.0
Percent Expressed
25
50
75
C
uv_response_dn
e2f_targets
myogenesis
myc_targets_v2
fatty_acid_metabolism
kras_signaling_dn
spermatogenesis
dna_repair
myc_targets_v1
coagulation
cholesterol_homeostasis
hypoxia
epithelial_mesenchymal_transition
glycolysis
apical_junction
apical_surface
angiogenesis
estrogen_response_late
estrogen_response_early
oxidative_phosphorylation
kras_signaling_up
pancreas_beta_cells
xenobiotic_metabolism
interferon_alpha_response
mtorc1_signaling
interferon_gamma_response
complement
pi3k_akt_mtor_signaling
il6_jak_stat3_signaling
apoptosis
tnfa_signaling_via_nfkb
adipogenesis
allograft_rejection
reactive_oxygen_species_pathway
il2_stat5_signaling
inflammatory_response
androgen_response
notch_signaling
wnt_beta_catenin_signaling
uv_response_up
p53_pathway
peroxisome
tgf_beta_signaling
unfolded_protein_response
mitotic_spindle
bile_acid_metabolism
heme_metabolism
g2m_checkpoint
hedgehog_signaling
protein_secretion
M0
M1
M2
1
0.5
0
-0.5
-1

FigureS7

(A) UMAP plot of macrophage colored by cell subtype. Each dot denotes a single cell.

(B) Dot plot of the expression levels of marker genes for macrophage subtypes defined in(a)Dot size corresponds to the percent of expressing cells, and dot color indicates the average expression levels.

(C) Heatmap showing differences in hallmark pathway activities scored per cell with GSVA among different macrophage subtypes.

Supplementary table 1 Details about the 13 samples used in scRNA-seq

Mice model/Tissue	ID	Group
Chrna5 KO mice/skin (KO house)	GSM5795803	KO group
Chrna5 KO IMQ mice/skin (koIMQ house)	GSM5795801	KO IMQ group
IMQ mice /skin (IMQ house)	GSM5795800	IMQ group
IMQ mice /dermis	GSM4547483	IMQ group
IMQ mice /skin	GSM5024748	IMQ group
IMQ mice/ skin	GSM5024749	IMQ group
IMQ mice treated with SHP099/skin	GSM5024750	SHP099 group
IMQ mice treated with SHP099/skin	GSM5024751	SHP099 group
WT mice/skin (WT house)	GSM5795802	WT group
WT mice/dermis	GSM4547481	WT group
WT mice/epidermis	GSM4547482	WT group
WT mice/skin	GSM5024746	WT group
WT mice/skin	GSM5024747	WT group

Supplementary table 2 Antibodies used in Fluorescence immunohistochemistry

Antibodies	Company	Cat No.	Dilution ratio
E-cadherin Monoclonal antibody	roteintech	0335-1-Ig	1:400
Vimentin Polyclonal antibody	Proteintech	0336-1-Ap	1:200
-conjugated Goat Anti-Rabbit IgG(H+L)	Proteintech	SA00013-2	1:500
– conjugated Goat Anti-Mouse IgG(H+L)	Proteintech	SA00013-3	1:500
Mounting Medium With DAPI	Abcam	ab104139	
Normal Goat Serum For Blocking	CWBio	CW0130	

Supplementary table 3 The primer sequences of qRT-PCR

Target	Forward Sequence (5’–3’)	Reverse Sequence (5’–3 ’)	Size (bp)
Human-Vim	TGCCGTTGAAGC TGCTAACTA	CCAGAGGGAGT GAATCCAGATTA	248
Human-E-cad	AAAGGCCCATTT CCTAAAAACCT	TGCGTTCTCTATC CAGAGGCT	172
Human-GAPDH	GGAGCGAGATCC CTCCAAAAT	GGCTGTTGTCAT ACTTCTCATGG	197
