# Supplementary figures and images for: Single-cell RNA-seq reveals keratinocyte and fibroblast heterogeneity and their crosstalk via epithelial-mesenchymal transition in psoriasis
Source: Cell Death Dis. 2024 Mar 12;15(3):207. doi: 10.1038/s41419-024-06583-z (PMC10933286; doi:10.1038/s41419-024-06583-z)

original

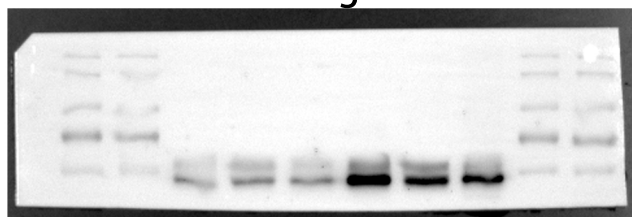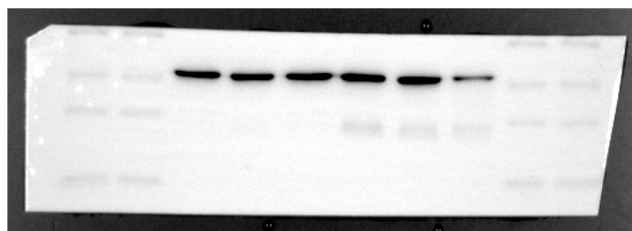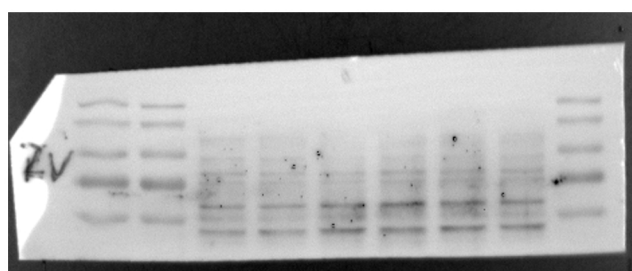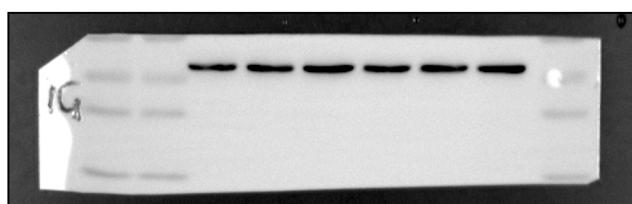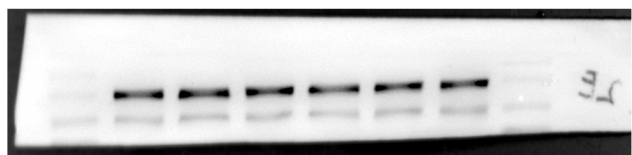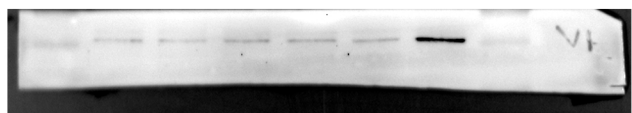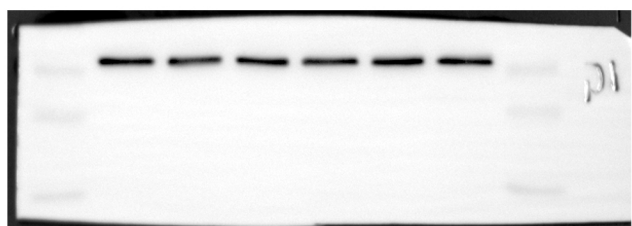

final

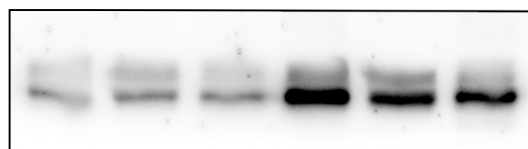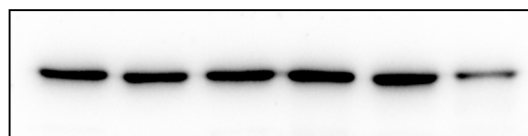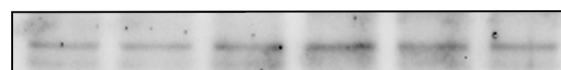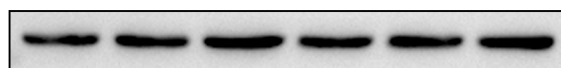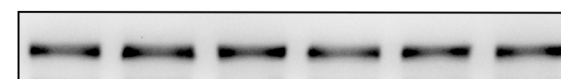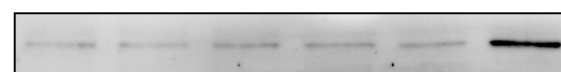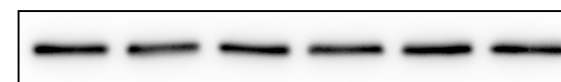

original

final

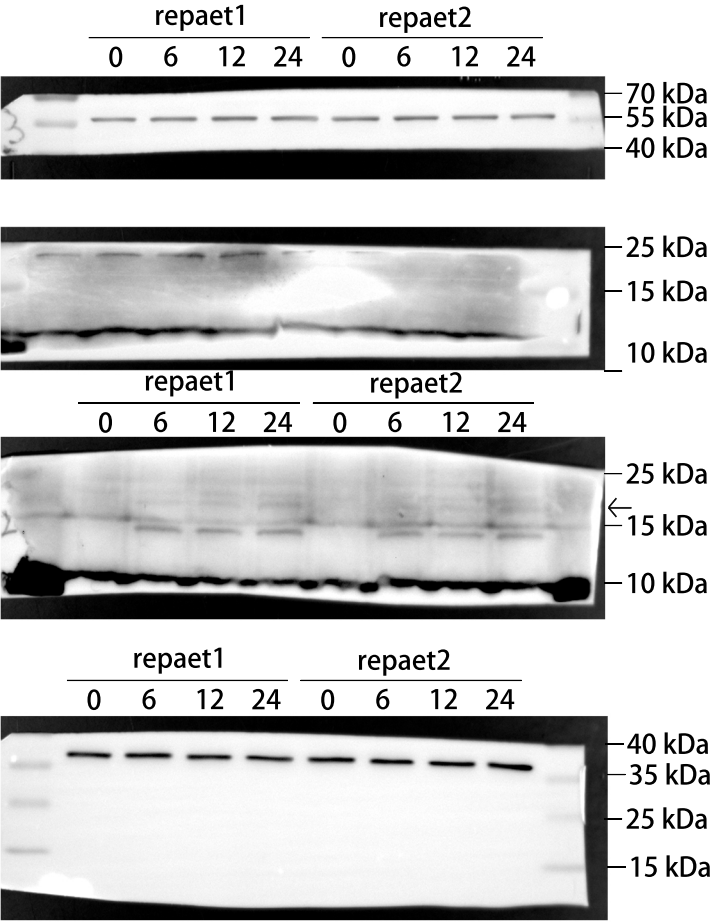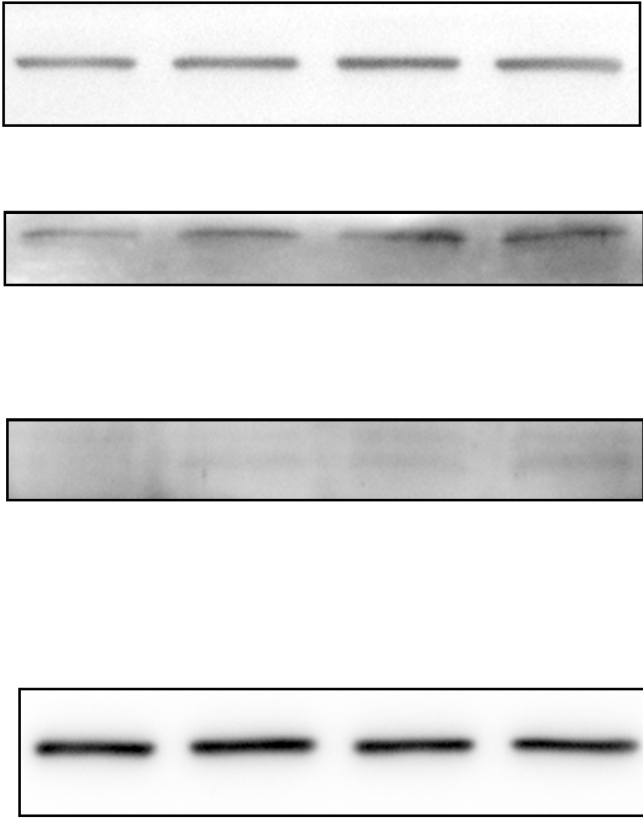

Supplement: Supplementary file 2 — Raw images of western blot [file 41419_2024_6583_MOESM2_ESM.pdf]
